# FLASH Radiotherapy: Benefits, Mechanisms, and Obstacles to Its Clinical Application

**DOI:** 10.3390/ijms252312506

**Published:** 2024-11-21

**Authors:** Lina Alhaddad, Andreyan N. Osipov, Sergey Leonov

**Affiliations:** 1 Department of Environmental Sciences, Faculty of Science, Damascus University, Damascus P.O. Box 30621, Syria; lina.alhaddad@damscusuniversity.edu.sy; 2 N.N. Semenov Federal Research Center for Chemical Physics, Russian Academy of Sciences, Moscow 119991, Russia; 3 Moscow Center for Advanced Studies, Kulakova Str. 20, Moscow 123592, Russia; leonov.sv@mipt.ru; 4 State Research Center–Burnasyan Federal Medical Biophysical Center of Federal Medical Biological Agency (SRC–FMBC), Moscow 123098, Russia; 5 CANDLE Synchrotron Research Institute, 31 Acharyan, Yerevan 0040, Armenia; 6 Institute of Cell Biophysics, Russian Academy of Sciences, Pushchino 142290, Russia

**Keywords:** ionizing radiation, conventional radiotherapy, FLASH radiotherapy, ultra-high dose rate

## Abstract

Radiotherapy (RT) has been shown to be a cornerstone of both palliative and curative tumor care. RT has generally been reported to be sharply limited by ionizing radiation (IR)-induced toxicity, thereby constraining the control effect of RT on tumor growth. FLASH-RT is the delivery of ultra-high dose rate (UHDR) several orders of magnitude higher than what is presently used in conventional RT (CONV-RT). The FLASH-RT clinical trials have been designed to examine the UHDR deliverability, the effectiveness of tumor control, the dose tolerance of normal tissue, and the reproducibility of treatment effects across several institutions. Although it is still in its infancy, FLASH-RT has been shown to have potential to rival current RT in terms of safety. Several studies have suggested that the adoption of FLASH-RT is very limited, and the incorporation of this new technique into routine clinical RT will require the use of accurate dosimetry methods and reproducible equipment that enable the reliable and robust measurements of doses and dose rates. The purpose of this review is to highlight the advantages of this technology, the potential mechanisms underpinning the FLASH-RT effect, and the major challenges that need to be tackled in the clinical transfer of FLASH-RT.

## 1. Introduction

Radiotherapy (RT) is required by 60–70% of tumor patients during their treatment [1]. Currently, there is no optimal RT method, and it triggers damage to normal tissue, limiting its use in tumor treatment [2]. The potential biological effects of ionizing radiation (IR) dose to organs at risk and target volumes are a dominant dose-limiting constrain in RT [3]. Therefore, finding ways to alleviate damage to the surrounding healthy tissue has always been a specific topic of interest in RT research. While contemporary in vitro and in vivo preclinical studies continue to report propitious normal tissue-sparing phenomena, systematic toxicity data are still needed [4]. The limitations of conventional RT (CONV-RT) can potentially be overcome by an emerging novel tool known as FLASH-RT [5]. Ultra-high dose rate (UHDR) IR has emerged as an auspicious RT approach to efficiently minimize potential damage load on normal tissue without sacrificing tumor control efficacy [6]. From the late 1950s on, numerous studies have shown an increased normal cell radioresistance when using UHDRs both in vivo [7] and in vitro [8,9,10]. FLASH-RT is typically defined as a non-invasive external RT technology involving the delivery of UHDRs to the target volume at several orders of magnitude higher than those used in current clinical CONV-RT (≥40 Gy/s vs. 1–4 Gy/min, respectively) for an extremely short duration of time [2,11,12,13]. Tumors located in the thoracic and upper abdomen areas, including the lungs, breast, liver, pancreas, and esophagus, often move significantly during breathing. Respiration-induced tumor motion has been shown to cause uncertainties in CONV-RT for these areas and is seen as a major obstacle in achieving precise dose distributions [14]. The short treatment delivery times used in FLASH-RT, often (<0.1 s), have the added value of reducing treatment delivery uncertainties triggered by the intra-fraction motion [15]. However, a safe application of this technology requires a sufficiently fine temporal resolution to handle UHDRs of FLASH-RT during the patient’s breathing cycle intervals, posing severe constraint on any FLASH-RT effect study [16]. Preclinical FLASH-RT in animal studies can be performed in small volumes and/or superficial targets through dedicated experimental facilities with a high IR output or modification of pre-existing RT systems, including synchrotron sources producing kilovoltage X-ray beams [17], proton beamlines [18], specialized electron linear accelerators (LINACs) [18], and converted clinical LINACs [19,20]. These systems allow the entire RT treatment, or large doses per fraction, to be delivered within a short time window, less than a few tenths of a second, compared to several minutes for CONV-RT [19,21,22]. It has been demonstrated that given both the potential impact of FLASH-RT to freeze physiological motion and its non-toxic radiobiological effect, it has the potential to be an evolutionary step in tumor treatment [15]. Precisely performed, this would keep the treatment margins as small as possible, and thus, smaller volumes of normal tissue would be needlessly irradiated [15]. RT has been found to induce indirect DNA damage through reactive oxygen species (ROS) generation, although increased radical-radical interaction resulting from enhanced ionization from UHDRs leads to less normal tissue toxicity. It has been reported that at UHDRs, the radiolysis of water expediting oxygen consumption before the re-diffusion of oxygen through tissues can maintain the microenvironmental oxygenation [2,23]. Another hypothesis is that the levels of DNA damage induced by FLASH-RT are lower than those following the CONV-RT dose rate [11,24,25,26]. Multiple in vitro studies have suggested that sub-millisecond pulses of IR elicit less genomic instability than continuous, protracted IR at the same total dose [12]. Furthermore, many authors have expected that immune responses could have a defining role in the FLASH-RT effect [27]. Previous studies have shown that FLASH-RT results in stark reductions in both immune response activation and chromosomal aberrations [13,28] The success of planned FLASH-RT clinical trials examining these and other hypotheses is based on the accuracy, precision, and consistency of UHDR technology implementation [29].

Differences in the extent of the beneficial FLASH-RT effect on normal tissues have been demonstrated across various studies, with some indicating that FLASH-RT may have deleterious impact or no observed sparing compared to CONV-RT [4,30,31].

Ongoing and planned FLASH-RT clinical trials have been designed to test the UHDRs deliverability and safety, with the examination of the effectiveness of long-term tumor control, tolerance dose of normal tissue, the trustworthiness of treatment effects across different institutions, and the safety and effectiveness of combined chemo-RT treatment models. This review provides readers with a better understanding of the differential effect of FLASH-RT in tumor tissue vs. normal tissue and summarizes mainstream hypotheses of the FLASH-RT effect. Moreover, it underscores the challenges and complexities of integrating this novel approach into the clinic.

## 2. FLASH-RT Definition and History

In recent years, FLASH-RT has gained momentum not only due to the beneficial effect on treatment duration but particularly for the promising reduction of normal tissue toxicity as shown in in vivo studies [12,32,33]. It has been shown that most patients receiving RT suffer from severe and long-term side effects [34]. In addition, it has been reported that if the RT dose needed to cure the tumor exceeds the normal tissue tolerance, the cure cannot be attained [34]. The recent surge of attention to UHDR “FLASH-RT” has opened a deluge of investigations trying to define, substantiate, exploit, and rationalize this interesting phenomenon [35]. FLASH-RT refers to UHDR delivery of therapeutic IR doses within a fraction of a second to a microsecond, as opposed to minutes with CONV-RT [35], which requires more robust beam delivery validation, advanced dosimetry scheme (incorporating dose rate data into RT planning), patient positioning, quality assurance, and safety interlocks [36]. At these dose rates, IR-induced normal tissue toxicity has been shown to be greatly reduced due to the fast deposition of IR energy that occurs too rapidly to maintain adequate oxygenation [37]. Based on a rigorous preclinical rationale, FLASH-RT has been shown to be able to markedly increase the normal tissue tolerance, termed the “FLASH-RT effect” [2]. The FLASH-RT effect can also be visualized as a dose-modifying factor (DMF) or dose-reduction factor—that is, the ratio of FLASH-RT to CONV-RT doses that achieve the same assay endpoint under the same physiological and anatomical conditions. The DMF has been shown to be greater than unity because FLASH-RT enhances normal cell sparing [38]. Across several studies, FLASH-RT generally has been shown to provide better protection in various normal tissues, with dose-modifying factors of 1.1 to 1.8, depending on endpoint and model species used [12,13,15,39,40]. Many studies have found that ultra-fast treatment delivery can be sufficient to circumvent organ motion-related problems [41,42]. It is unclear what parameters of UHDR, encompassing potential dose and/or dose rate threshold, are optimal to generate the FLASH-RT effect. The FLASH-RT effect has been mainly investigated in electron beams [18,43], but preclinical trials in X-ray [17], as well as in carbon ion beams and proton, have increased in number [39,44,45]. It has been pointed out that in vitro investigations do not substitute for in vivo validation of the FLASH-RT effect [46]. The FLASH-RT effect has been investigated using bacteria, non-tumor mammalian cell cultures, and human lymphocytes [32]. Early studies carried out in the field of UHDR-RT have taken place in an in vitro setting, primarily because of technical complications with delivering such dose rates with sufficiently large field sizes for animal irradiation.

Modeling approaches are useful for explaining the differential FLASH-RT effect. Accordingly, several theoretical frameworks involving the role of oxidation–reduction biology and tissue oxygenation have been reported [47,48]. There is a rich history of formerly published papers characterizing the oxygen-concentration dependence of the FLASH-RT effect [37,49,50]. For normal cells, FLASH-RT has been shown to increase the survival rate due to the oxygen enhancement ratio (OER) effect, by depleting them of oxygen [51].

Despite the spike in FLASH-RT interest in recent years, the advantages of using UHDR-RT and its effect were reported as far back as the 1950s–1970s [8,52,53]. In 1966, Hornsey and Alper investigated the survival of mice treated at a dose rate between 0.2 and 500 Gy/min following total body irradiation (TBI). They observed a reduction in survival with increasing dose rate, albeit reaching a plateau, and even a modest increase in survival at dose rates exceeding 100 Gy/min. Additionally, they deduced that at UHDRs, local oxygen depletion would start to play a role in diminishing the effectiveness of IR [53]. In 1967, Town described the difference in the survival of HeLa cells exposed either to one or two pulses of 14 MeV electrons in aerobic suspensions [54]. These investigators thought that the biphasic nature of the single-pulse HeLa cells’ survival curve was triggered by the removal of oxygen from the relevant site inside the cell by its reaction with the intermediates produced during IR. In this system, they demonstrated that the radiobiologically relevant oxygen was removed by a dose of about 9 Gy during the 1.3 μs pulse [54]. In 1968, Todd et al. found no decrease in the sensitivity of cultured human kidney cells exposed to 10 MV X-rays delivered in 30 ns pulses, up to a dose level exceeding 10 Gy in air and 30 Gy under hypoxic conditions [55]. In 1969, Oliver suggested that much more experimental work was needed and, in particular, that it would be very interesting to elucidate the results of irradiating cells under hypoxic conditions at UHDRs [56]. In the same year, Berry et al. suggested a dose-rate mechanism, including radical-radical interactions. They reported some interesting results that displayed an apparent decrease in the effectiveness of pulsed UHDRs X-ray in abolishing the reproductive potential of Chinese hamster and HeLa cells in vitro compared to IR delivered at lower dose rates [57]. Their published observations did not demonstrate a reduced oxygen effect under aerated conditions in the dose range of 1 to 10 Gy and at dose rates of 10^9^ to 10^10^ Gy/s [57]. The UHDR skin and intestine sparing effect was shown in the 1970s [7,37,58]. In 1970, Nias et al. reported survival curves of HeLa cells irradiated with single 10 ns electron pulses under aerated and anoxic conditions [59]. In 1973, Berry compared the effects of dose rate from protracted, continuous IR compared to UHDR from pulsed accelerators [52]. In 1974, Field and Bewley showed that irradiation of the hind feet of anaesthetized rats with single doses of 7 MeV electrons at 67 Gy/s caused a very slight change in skin shape and induced less severe adverse skin reactions, such as, erythema, swelling, skin surface breakdown, and moist desquamation, in the short and long term compared to those exposed to either 1 or 0.03 Gy/s. Furthermore, they reported that UHDR triggered oxygen depletion in rat skin, as it did in mouse skin [58]. In 1969, Prempree et al. demonstrated a reduction in the yield of dicentric aberrations from 15% to 10% for human lymphocytes exposed to 2 Gy of X-rays, delivered in a 2-ns pulse, compared to a CONV-RT dose rate of 1 Gy/min [60]. On the basis of the results of an additional experiment in 1977, the frequencies of dicentrics were shown to be independent of dose rate, although a somewhat wider range of dose rates was tested [61]. In 2009, Martin published a paper that generated considerable excitement about the therapeutic potential of the laser-accelerated subatomic particles. The author pointed out that this fledgling technology offers greater tissue-sparing precision of proton and carbon ion beams RT than does CONV-RT [62]. In 2011, Schmid et al. determined the production of IR-induced chromosomal aberrations type in monolayers of human-hamster hybrid cells (A_L_ cells) by measuring the relative biological effectiveness (RBE) values for continuous and pulsed 20 MeV protons relative to 70 KV X-rays as the reference IR [63]. The term “FLASH-RT” was first coined in 2014 by Favaudon et al. in Lausanne University Hospital [12]. They showed that the use of single doses of FLASH-RT minimized the appearance and severity of early and delayed complications affecting normal lung tissues and allowed complete mouse lung tumor removal [12]. In 2017, Montay-Gruel et al. showed for the first time a preservation of spatial memory in mice 2 months after 10 Gy whole brain IR (WBI) FLASH-RT with average dose rates above 100 Gy/s, whereas 10 Gy at a CONV-RT dose rate (0.1 Gy/s) totally impaired spatial memory [13]. In 2018, Patriarca et al. conducted the first in vivo proton FLASH-RT experiment on mouse lung using a medical proton cyclotron to deliver a spread-out Bragg peak proton beam to cover the deep target homogeneously; proton FLASH-RT dose rates (>40 Gy/s) with doses on the order of 20 Gy were achieved, which were impossible with the CONV-RT clinical mode [45]. The first proof of concept of X-ray FLASH-RT effect was first reported by Montay-Gruel et al. in 2018. They observed that a 10 Gy delivered at UHDR synchrotron light source-generated X-rays prevented memory disturbance, elicited less reactive astrogliosis, and decreased hippocampal cell-division impairment in mice [17]. In 2018, Smyth et al. reported the first in vivo dose-equivalence data for microbeam RT, synchrotron broad-beam RT, and CONV-RT. They presented systematic IR-induced toxicity data for a range of mouse organs, which can serve as a reference point for future preclinical studies [4]. They observed no significant radiobiological differences in median toxic dose (TD50) values between UHDR synchrotron broad-beam RT and CONV-RT [4]. In clinical practice, IR dose rate delivery had not been perceived as a significant, manipulable RT variable. In 2019, Vozenin et al. sought to change this paradigm by extending their previous in vivo preclinical study in mouse models [12] to confirm the clinical safety and efficacy of FLASH-RT in mini-pig and cat tumor patients [15]. This novel approach fused new radiobiology and technological advancements by delivering electron beams at FLASH-RT dose rates around 300 Gy/s, yielding fascinating results [15]. In 2020, Soto et al. published the first report of a dose-response study on skin toxicity in mice that received a single fraction hemithoracic FLASH-RT [64]. Also, in that year, Jin et al. reported the first computational model that clarified a very strong sparing effect on circulating immune cells by single-fraction high dose FLASH-RT [65]. The first clinical study on the safety and feasibility of FLASH-RT in canine tumor patients with microscopic residual disease and spontaneous superficial solid tumors, using modified clinical LINAC electron beams, was presented by Konradsson and colleagues in 2021 [66]. A phase I clinical dose-escalation trial in tumor-bearing dogs with metastatic melanomas (IMPULSE trial), using 10 MeV electron beams from a modified Elekta Precise LINAC at different dose levels (15 to 35 Gy), began in March 2020 at the Centre Hospitalier Universitario Vaudois in Lausanne, Switzerland. The calculated depth dose curves showed the typical features of electron beams, with a high surface dose and a rapid dose fall-off beyond dose maximum (R_80_-value) due to scattering and beam energy loss. This study also discovered that a single fraction electron FLASH-RT (430–500 Gy/s) could be a viable treatment option for canine superficial and subcutaneous tumors located at a depth of 2–3 cm within the tissue [66]. Hence, deep-seated tumors were not included in this trial. In 2020, Diffenderfer et al., announced that the proton FLASH-RT system at the University of Pennsylvania was able to deliver a proton beam at a dose rate of 60–100 Gy/s and provided the first evidence of proton FLASH RT-mediated normal tissue radioprotection in mice [39]. The first report on the application of high-energy X-rays with FLASH-RT and its corresponding implementation in vivo was reported by Gao et al. in 2022 [67]. In 2022, Konradsson et al. published the first report that showed no difference in long-term tumor control rates, tumor response, acute and late toxic adverse effects between hypofractionated FLASH-RT (70–90 Gy/s) and CONV-RT (~8 Gy/min) in immunocompetent glioblastoma multiforme (GBM)-bearing rats for any of the dose levels tested at any of the investigated time points [68]. Tashiro et al. successfully performed the first in vitro human cell experiments with FLASH-RT carbon ions (1–3 Gy at 13 or 50 keV/μm beams) at dose rates of 96–195 Gy/s. There was no sparing effect observed on the growth suppression and cellular senescence of lung fibroblast cells HFL1 5 days post-IR, or on the clonogenic survival of salivary gland tumor line HSGc-C5 7 days post-IR with both linear energy transfer (LET)13 and LET50 in FLASH-RT [69].

To date, and to our knowledge, there have been two published in-human case studies of the clinical application of FLASH-RT: (1) A 75-year-old patient with a multiresistant T-cell cutaneous lymphoma [2]. This first FLASH-RT treatment was shown to be safe and feasible with a favorable outcome both on normal skin and tumor [2]; (2) A first-in-human phase I clinical proton FLASH-RT trial using a single fraction dose of 8 Gy was described by Mascia et al. in 2023 [36]. In this case, treatment of 10 patients aged ≥ 18 years with up to three painful bone metastases in the extremities (excluding wrist, hand, and feet metastases) using a single-transmission proton beam (FAST-01 study, NCT04592887) was approved by the Cincinnati Children’s Hospital Institutional Review Board and the Food and Drug Administration in the United States [36]. Data from this trial were collected from 3 November 2020 to 28 January 2022. In this nonrandomized clinical human study, safety data revealed that proton FLASH-RT was clinically efficient, feasible, and its safety was supported by the minimal intensity of related adverse effects. However, the effectiveness of proton FLASH-RT treatment (>40 Gy/s) for pain relief at the treated metastatic sites appeared to be similar to that of the CONV-RT dose rate (roughly 0.03 Gy/s) photon RT system [36,70].

## 3. Comparing FLASH-RT to CONV-RT and Stereotactic Body RT

The approach used in CONV-RT is to give the tumor patients a low total dose with a low dose rate over several short sessions, “fractions”, which are separated by a time interval of at least 1 day. This method has been shown to be based on the knowledge that healthy cells have a greater chance of survival if doses are small and divided into fractions separated in time. A total dose of 60–66 Gy in 1.8 to 2 Gy fractions has been found to be the highest tolerated dose that can be safely delivered to tumor cells [71]. It has been shown that splitting the total dose into smaller fractions is based on the time scales of biological responses. It has been found that tumor cells tend to have inferior DNA repair capabilities, increasing the therapeutic effect of the treatment, whereas normal cells have been found to be able to repair DNA better and survive between doses [72]. It has been shown that delivering higher IR doses in fewer days leads to catastrophic health consequences for the patients [22,73]. As mentioned previously, FLASH-RT involves the ultrafast dose delivery at dose rates generally several thousand times larger than the ones currently used in routine clinical practice [43]. Although FLASH-RT has been initially characterized using an average dose rate of ≥40 Gy/s and a pulse amplitude of ≥10^6^ Gy/s, its full definition is more complicated and includes many interconnected physical parameters, such as repetition frequency, pulse width, pulse number, and total exposure duration [11,22]. Other beam parameters between the various beams should always be precisely monitored and controlled, including, among others: energy, total dose, average dose rate (the total dose/the entire delivery duration), instantaneous dose rate (the dose per pulse/the pulse duration), source-to-surface distance, field size, and beam-on time [74,75]. A growing body of evidence shows that the physical parameters of importance can differ depending on the macro- and microstructure of the beams, making the identification of common values between X-ray, electron, and proton FLASH-RT challenging [74,75]. For normal cells, FLASH-RT has been shown to reduce ROS levels, whereas CONV-RT significantly increases them [76]. A comparison between CONV-RT and FLASH-RT can be found in Table 1.

Stereotactic body RT (SBRT) enables the administration of high IR doses per fraction (6–30 Gy) to small, precisely defined targeted volumes (<5 cm) using a highly conformal approach; however, the steep dose gradients require careful attention to the healthy tissues nearby [77]. The standard dose of SBRT (range: 48–66 Gy) can be given in a short period, like five sessions or fewer in 2 weeks or less, as opposed to the several doses over weeks (6–7 weeks) needed in CONV-RT [78,79]. At present, SBRT can be conducted using different devices, including LINACs, Gamma Knife, Cyberknife, or Tomotherapy [80]. Despite the fact that the high doses/fraction associated with SBRT are unlikely to surpass the dose thresholds for a single beam, SBRT could still represent one of the most appropriate situations for FLASH-RT of deeply seated tumors [81]. SBRT has been used in patients with medically inoperable early-stage primary and oligometastatic tumors [82]. Prior research has demonstrated outstanding tumor management effectiveness of SBRT [83]. For example, A dose of 30 to 34 Gy in a single-fraction SBRT has been reported to be effective for treating peripheral early-stage non-small lung cancer cells [84]. One issue regarding the use of the SBRT technique is their possible vulnerability to motion caused by breathing, which could result in dosimetric uncertainty and differences between the planned and actual doses delivered. This technique requires superior motion management and imaging guidance systems, which might be lacking or expertise may not be present in every healthcare system [85]. The use of SBRT is frequently restricted due to dose-limiting toxicities affecting organs at risk when compared to FLASH-RT [86]. Numerous conformal SBRT studies have indicated that treatment-related toxicity complications are clinically serious [87]. An additional limitation of SBRT is the extended treatment duration linked to patient positioning and IR delivery. Patient setup time can range from 22 min [88] to 100 min for treatment delivery, depending on the equipment and dosage used [89]. Extended treatment durations greatly enhance the likelihood of intrafraction motion and mistakes [90]. Unlike FLASH-RT, SBRT is affordable and possibly enhances patient accessibility.

## 4. The Motives Behind FLASH-RT

**Figure 1:**
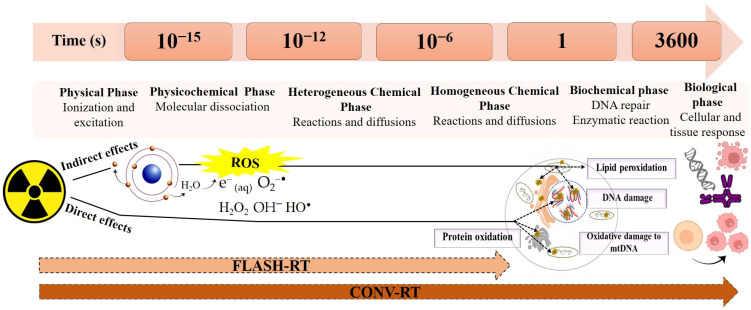
Schematic overview of early physical, chemical, and biological phases following IR of cells and tissues. The time scale has been proposed by Vozenin et al. [32].

The overall time for the total dose delivery using FLASH-RT is much faster than the time needed for repair of DNA and other biological processes (minutes), but is still significantly slower than the radio-chemical process of radiolysis of water molecules (10^−16^–10^−7^ s) [91]. CONV-RT interferes with the chemical and biological steps, while FLASH-RT does not interact with the biochemical steps (Figure 1).

**Figure 2:**
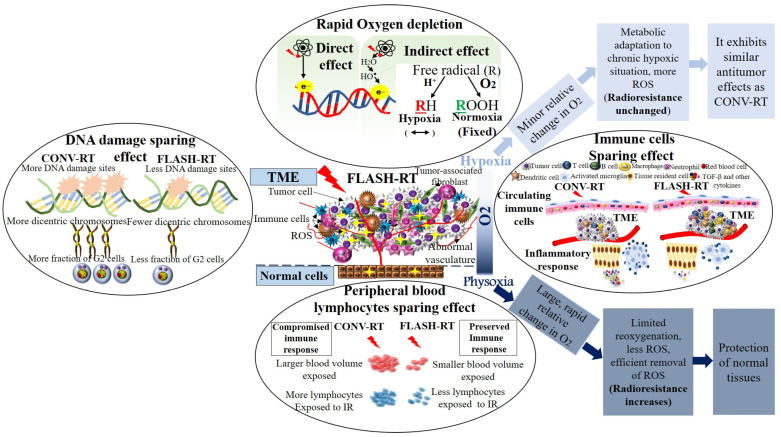
Biological benefits of FLASH-RT.

Multiple lines of evidence have revealed that FLASH-RT significantly reduces the incidence and severity of IR-induced normal tissue toxicity compared to CONV-RT (Figure 2) [11,12,13,64]. It has been reported that the extremely short exposure time of FLASH-RT can be exploited for better targeting of mobile tumors, which move due to respiratory motion [11,22,92]. The timescales over which oxygen consumption and rediffusion occurs have been shown to be important, because the underlying assumption is that the ultra-rapid delivery of IR doses is able to significantly deplete oxygen within the normal tissue before it can replenish, creating a temporarily hypoxic environment that consequently confers transient radioresistance [5,7,10,11,12,58,93,94,95,96,97]. It has been concluded that diffusion of oxygen into depleted oxygen sites following FLASH-RT occurs on the scale of 10^−3^ s [98].

Recent outcomes of animal experiments have shown that FLASH-RT triggers a similar anti-tumor efficacy as compared to CONV-RT at isodoses in lung, head and neck, and breast tumor models [3,12,23]. The iso-efficacy of CONV-RT/FLASH-RT on tumors has often been deduced from the lack of a significant difference in tumor growth kinetics following IR [99]. Many in vitro studies have suggested that sub-millisecond pulse IR induces less genomic instability than protracted continuous IR at equivalent total doses [12]. Some preclinical studies have indicated the nonfibrogenic character of FLASH-RT response [12].

## 5. FLASH-RT Beam Modalities

The FLASH-RT mode is generally defined by the overall treatment time, the instantaneous dose rate, and the average dose rate of the whole irradiation process [46,100]. However, parameters such as the total dose delivered, IR source, as well as pulse-related factors like pulse size, dose and dose rate per pulse, and the frequency of the pulse delivery, should also be carefully taken into account [11,32,35,50,101]. Unfortunately, these details are often overlooked for specific studies. They are essential when trying to thoroughly evaluate if the beam-delivery conditions are adequate to observe the FLASH-RT effect.

Recent studies have shown that clinical LINACs can be modified to deliver electrons at FLASH-RT dose rates [19,43]. To date, most FLASH-RT was conducted with a 3–18 MeV electron beam achieved by either LINACs originally designed for non-destructive examination/evaluation, such as the Oriatron eRT6 [18,102] or by modified clinical LINACs operating in the electron mode [19,103]. Electron beam FLASH-RT is unlikely to revolutionize RT owing to the simple fact that the advantages of this technique are only applicable to superficial and shallow-seated tumors, such as skin tumors. Therefore, other techniques and treatment devices are required for FLASH-RT to be clinically proper for more than intraoperative RT or superficial RT. Other potential options include very high energy electrons (VHEE) or the use of proton or photon beam-based FLASH-RT. In 2015, Bazalova-Carter et al. optimized a rapid RT treatment planning using VHEE scanning pencil beams in the range of 60–120 MeV [104]. The authors showed that VHEE plans led to superior or similar dose distributions for lung, prostate, and pediatric cases compared to clinical volumetric modulated arc therapy (VMAT) plans [104]. An additional advantage is that dose distributions generated by VHEE beams are less sensitive to tissue heterogeneity than those generated by proton and photon beams [104].

Some studies have focused on producing the FLASH-RT effect using X-ray beams. Even if the RBE of X-rays and electrons at CONV-RT is identical, the possibility of MV X-rays to observe the FLASH-RT effect would open up the treatment possibilities for deep-seated tumors, thanks to their higher penetration profile compared to electrons. UHDR X-ray beam production is challenging, as it is limited by electron heat deposition in the target [74]. Implementation of high-energy X-rays with FLASH-RT has been shown to be advantageous because of its small divergence, deep penetration, and cost-effectiveness [67]. Gao at al. showed the possibility of observing the FLASH-RT effect in a mouse model with high-energy X-rays at average dose rates of around 750 Gy/s, and (spatial)-instantaneous dose rates of >10^6^ Gy/s [67]. There are two major processes leading to the production of UHDR X-ray beams, namely bremsstrahlung (braking) IR and synchrotron IR [74]. Moreover, pulsed UHDR X-rays can be generated by high-temperature plasma [74]. High-powered CONV-X-ray tubes have been shown to be suitable for in vitro FLASH-RT research and dosimetry experiments [105]. In 2019, Bazalova-Carter and Esplen showed by Monte Carlo model and indirect measurements that an unfiltered 160 kVp continuous X-ray beam delivered by the Comet 3 kW MXR-160/22 and 6 kW MXR-165 X-ray tubes could provide dose rates of >100 Gy/s on the surface of a plastic water phantom located right against the X-ray tube surface [105]. Furthermore, Cecchi et al. modified a CONV-X-ray tube-based system with a beam shutter for in vitro UHDR delivery and reached dose rates of up to 118 Gy/s [106]. It has been proposed that high-activity radioisotopes could lead to UHDR-IR (γ-ray emitting nuclides) [74]. Proton therapy has been shown to be better at conforming doses than electron beams, making it a suitable option for treating different types of tumors [107]. Proton FLASH-RT has been shown to offer enhanced RBE in the Bragg peak compared to electron and photon-based RT when treating prostate tumors, head and neck tumors, and pediatric tumors, among others [108,109,110,111,112,113]. Both Buonanno et al. [114] and Beyreuther et al. [30] examined the utilization of proton beams in administering FLASH-RT. Furthermore, Girdhani et al. conducted a study comparing FLASH-RT to CONV-RT with proton beams to evaluate potential lung-sparing effects [115]. The initial clinical application of proton FLASH-RT has employed a high-energy proton transmission beam that travels directly through patients [36]. However, this forfeits a crucial benefit of proton beam therapy, as protons with carefully selected energies halt right after the tumor. Transmission proton beams deposit undesired doses of IR to organs at risk beyond the target due to the exit dose received [116]. In contrast to scattering-based methods, pencil beam scanning is the leading method for delivering protons and is the most common treatment approach in proton therapy. It achieves better dose conformity for clinical targets of various sizes by using pencil beam proton spots instead of a uniform scattered proton beam [117]. A novel method for proton therapy was created at the New York Proton Center for precise delivery of FLASH-RT using Bragg peaks [118]. By retracting the ranges of the most energetic proton beams and adjusting proton ranges to fit the distal target, the exit dose of proton beams can be removed to enhance protection for organs at risk while maintaining FLASH-RT dose rate delivery [118]. In order to reach UHDRs, the Bragg peak FLASH-RT approach uses the cyclotron’s highest energy output for the highest beam current. Cyclotron proton accelerators are presently the first and only clinical systems designed to treat deep-seated tumors with FLASH-RT dose rates; however, meeting the FLASH-RT conditions remains difficult even with these machines [116,119]. The biggest challenges faced by cyclotrons in administering FLASH-RT are associated with beam energy. Switching energy standards cannot be employed to provide proton Bragg peaks to multiple tissue layers in FLASH-RT due to beam losses and the time required to adjust the beam energy from the cyclotron, which can range from 80 ms to several seconds per alteration [107,120]. Current clinical Isochronous cyclotrons can provide beam currents up to 800 nA, while research systems can generate currents exceeding 100 μA. The degrader in the beam transport system changes the beam energy by modifying the physical thickness and/or density of the degrader material. Changing beam energy rapidly in FLASH-RT systems can be difficult, potentially requiring < 0.1 ms per energy step. Cyclotrons face a major challenge in creating degraders with less loss and improving energy selection for delivering beams with large acceptance, which in turn lowers the need for maximum extracted current [120]. While early proton FLASH-RT accelerator tests used synchro-cyclotrons, technical constraints may hinder their application in complete spot-scanning FLASH-RT systems. The repetition frequency and pulse structure of synchro-cyclotrons are not as compatible with FLASH-RT delivery needs as the steady continuous-wave lasers beams from isochronous cyclotrons [120]. The mini-ridge filter technique is also applicable for delivering conformal doses to the target. Ridge filters are static devices positioned on the beamline, so they do not need any additional modulation besides the rotating modulator [121]. This method utilizes pin- or blade-shaped range modulators to modify the pristine Bragg peak, creating a spread-out Bragg peak over a specific depth range without altering the beam energy extracted from the cyclotron. This ensures uniform dose distribution in one beam and enables the administration of a high dose to the tumor while minimizing exposure to nearby healthy tissue [122,123]. Over the past few years, there has been a notable rise in interest in ridge filters as a result of the increasing focus on FLASH-RT experiments for scheduling studies involving actual patient cases [120,124]. Evans et al. [125] and Kim et al. [121] carried out experiments on mice by employing ridge filters to generate spread-out Bragg peaks utilizing clinical synchrocyclotron and cyclotron proton therapy systems. Their findings provided initial validation of the possibility of combining the benefits of the Bragg peak with the tissue-sparing advantages of FLASH-RT. Pennock et al. assessed the proton FLASH-RT strategies for organ at risk sparing in eight patients with recurrent head-and-neck cancer: three from the oropharynx, two from the oral cavity, one from the sinonasal area, one from the nasopharynx, and one from the salivary gland [126]. Their results showed that Bragg peak proton FLASH-RT provided better organ preservation compared to transmission proton FLASH-RT, even though both techniques offered comparable coverage of target and FLASH-RT dose rates [126].

## 6. Effect of FLASH-RT

Any tumor could be managed with RT if an adequate dose was administered to all tumor cells. Despite advancements in technology for delivering physical treatments that enhance IR-dose conformity, normal tissues are inevitably encompassed in any IR field within the tumor volume. The main objective of RT is to maximize the IR dose to tumor cells while reducing the exposure to nearby normal cells or those in the IR’s path. This provides the best opportunity to treat the tumor while lowering the likelihood of side effects. The side effects induced by CONV-RT represent a major concern for non-target normal tissues, limiting the curative potential of RT for many tumors, particularly those located near critical organs, such as the lungs and heart [35]. The early definition of FLASH-RT has served as a starting point for discovering dose rates at which the FLASH-RT effect can be predicted, yet the mechanistic underpinnings underlying the FLASH-RT effect remain to be conclusively substantiated. The possible emergence of the FLASH-RT effect has lately spurred a surge of investigations attempting to describe and rationalize this phenomenon. Reports of sparing of adjacent normal tissue and anti-tumor efficacy at UHDRs have fueled the FLASH-RT field. Several studies have delved into the FLASH-RT effect using in vitro cell culture and in vivo animal models, as summarized in Table A3 and Table A4.

### 6.1. Normal Tissue Sparing

FLASH-RT has been shown to evoke vigorous, reproducible responses across multiple organ systems, including the skin [15,32,58,64], bone [70,127], brain [13,72,128,129,130], lung [12], bone marrow [131], intestine [33,132], and ovary [133], in various species, including rodents [12,13,17,24,58,72,128,129,131], zebrafish embryos [28,134,135], mini-pigs, cats [15,21], dogs [136], and humans [2,36] (summarized in Table A1).

A series of experimental studies have reported that the instantaneous oxygen depletion and subsequent low production of ROS levels during FLASH-RT constitute one of the major mechanisms able to change the entire biological cascade driving toxicity in adjacent healthy tissues [7,10,24,58,97,137]. An early study from Favaudon et al. presented the first side-by-side in vivo evidence of the FLASH-RT sparing effect. They exposed C57BL/6J mice to bilateral thoracic irradiation using either pulsed (≥40 Gy/s, FLASH-RT) or CONV-RT dose rate (≤0.03 Gy/s) in a single fraction, and monitored the response of (1) human laryngeal (HEp-2) and luminal breast (HBCx-12A) tumor xenografts in nude mice, and (2) syngeneic luciferase-positive (Luc+) TC-1 orthotopic lung tumor in mice to both irradiation modalities. All FLASH-RT irradiations were performed using a LINAC (4.5 MeV electrons). For CONV-RT, 200 kV X-rays were employed for irradiating HEp-2 tumor xenografts, while ^137^Cs γ-rays were used for irradiating the bilateral thorax of mice and HBCx-12A tumor xenografts. They found that a 17 Gy CONV-RT induced the activation of TGF-β signaling cascade and lung fibrosis in all the animals 24–36 weeks post-IR, whereas no complications developed by FLASH-RT below 23 Gy, and a 30 Gy FLASH-RT was required to induce comparable levels of fibrosis as observed following 17 Gy CONV-RT. Furthermore, they found that FLASH-RT minimized cutaneous lesions in severity and caused significantly less pneumonitis, and less acute apoptosis of pulmonary capillary endothelial cells, compared to CONV-RT [12].

Soto et al. observed that single-fraction hemithoracic FLASH-RT in mice with high doses of 30 and 40 Gy resulted in both lower severity and lower incidence of skin ulcerations 8 weeks post-IR compared to CONV-RT, whereas no ulceration was found in either CONV-RT or FLASH-RT at doses (≤20 Gy) [64]. Girdhani et al. showed that IR-exposure of mice to whole-thorax proton FLASH-RT (40 Gy/s) using a single dose of 15, 17.5, and 20 Gy resulted in a decreased incidence of IR-induced skin dermatitis and a significant decrease in lung fibrosis compared to conventionally treated animals (1 Gy/s protons) [115]. The results revealed that FLASH-RT using clinical proton beams might allow for sparing of normal tissue toxicity after IR, and the major pathways differentially regulated between the two treatments involved DNA damage and repair, immune, and inflammatory responses modulation [115]. Similarly, Cunningham et al. demonstrated that skin and plasma levels of TGF-β1, leg contracture, and skin toxicity were all significantly decreased following delivery of a single dose of 35 Gy proton pencil beam scanning FLASH-RT compared to CONV-RT group mice [127].

Levy et al. developed a preclinical IR platform for total abdominal FLASH RT (TAI-FLASH-RT) in mice. They observed that large single doses of 12–16 Gy TAI-FLASH-RT (216 Gy/s, electrons) produced less lethality from IR-induced gastrointestinal syndrome, spared gut function and intestinal epithelium integrity, and produced less early DNA damage and less apoptosis in intestinal crypt base columnar cells compared to TAI-CONV-RT (0.079 Gy/s, electrons) [33]. FLASH-RT has been shown to have minimal impact on neural [13], intestinal [132], and epidermal stem cells [15]. It has been reported that the mechanisms of FLASH-RT sparing of the intestinal crypts probably stem from the specialized tissue architecture of the intestine and the high proliferative capability of intestinal stem cells under injury conditions [138]. Diffenderfer et al. used syngeneic MH641905 cells (autochthonous pancreatic tumor) grafted in the flank of C57BL/6J mice. Tumors were irradiated with 230 MeV protons at FLASH-RT dose rates alongside CONV-RT dose rates irradiation. Their results showed that TAI-FLASH-RT at 15 Gy significantly decreased the loss of proliferating crypt cells compared to CONV-RT, and 18 Gy FLASH-RT inhibited the development of intestinal fibrosis as a long-term effect post-IR [39]. Ruan et al. investigated the effect of average dose rate and temporal pulse structure of FLASH-RT compared to CONV-RT on acute intestinal toxicity. They irradiated the whole abdomens of C3H mice with a single fraction to various doses, using a 6 MeV electron LINAC with single pulse FLASH-RT (2–6 × 10^6^ Gy/s) or CONV-RT (0.25 Gy/s). Their findings indicated that FLASH-RT at doses between 7.5 and 12.5 Gy led to considerable improvements in crypt survival, with a DMF of 1.1. In their study, sparing effect was observed for average dose rates of ≥280 Gy/s or higher. The fecal microbiome analysis showed that FLASH-RT triggered fewer changes to the gut microbiota composition 3.75 days post-IR compared to CONV-RT [139].

Many studies have reported that the typical RT protocols contribute to IR-induced neurocognitive dysfunction involving deficits in learning and memory, executive function, attention, and a multitude of abnormalities in mood that can be associated with cortical and hippocampal-based changes [13,32,140]. Similarly, Alaghband et al. reported the protective role of the FLASH-RT effect in the neurogenic niche in radiosensitive juvenile mice. They found that a single dose of 8 Gy WBI-CONV-RT (0.077 Gy/s, 6 MeV electrons) triggered considerable declines in different indices of cognitive-behavioral performance, reductions in the quantity of mature NeuN-positive and immature doublecortin-positive neurons, and augmented neuroinflammation within 2–4 months post-RT, adverse side effects that were not observed with the similar isodose delivered with 6 MeV electrons at the FLASH-RT dose rate (4.4 × 10^6^ Gy/s) [129]. Their data indicated the exciting clinical prospect of applying FLASH-RT in efforts to provide some long-expected relief for children who are diagnosed with medulloblastoma and are slated to undergo RT [129]. At another 6-month follow-up study, the same authors showed that IR-exposure of adult (10-week-old) mice to hypofractionated (3 × 10 Gy) WBI-FLASH-RT at an average dose rate of 5.6 × 10^6^ Gy/s preserved synaptic integrity and plasticity through multiple measures that were coincident with decreases in neuroinflammation (the levels of reactive CD68+ microglia) throughout certain brain areas (medial prefrontal cortex, ventral hippocampus) [141]. However, they did not find differences in the ultrastructure changes in presynaptic Bassoon and postsynaptic Homer-1 puncta bouton within the same brain areas in response to the IR-dose rate [141]. Simmons and colleagues demonstrated that the reduction of neurocognitive deficits after exposure of mice to a high single fraction dose 30 Gy of WBI-FLASH-RT at average dose rates of 200 or 300 Gy/s for 20 or 16 MeV electron beams, respectively, are related to less loss of hippocampal dendritic spine and less activation of microglial inflammation 10 weeks post-IR compared to 240 s CONV-RT delivery time with average dose rate of 0.13 Gy/s for 16 and 20 MeV electron beams [128]. Montay-Gruel et al. showed that a single dose of 10 Gy WBI-FLASH-RT, delivered with a dose rate exceeding 100 Gy/s in a mouse model, reduced behaviors characteristic of depression and anxiety, and did not induce deficits in fear extinction memory 6 months following irradiation compared to 10 Gy CONV-RT (0.07–0.1 Gy/s) [24]. Moreover, FLASH-RT attenuated neuroinflammation and preserved dendritic spine density and mature neuronal morphology 2 and 6 months post-IR [24]. In another preclinical study by the same authors, a pan-encephalic irradiation with a single dose of 50 Gy FLASH-RT (>50 Gy/s) did not elicit any macroscopic toxicity nor changes in GBM-bearing mice’s health, whereas 10 Gy WBI-CONV-RT (0.04 Gy/s) was the maximum tolerated dose for long-term follow-up. Moreover, their results showed that doses up to 20 Gy FLASH-RT preserved the neural stem cells pool in the subventricular zone 2 months following irradiation, while 10 Gy CONV-RT eradicated all the neural stem cells in the hippocampus [142]. The study by Montay-Gruel et al. using different irradiation schemes has helped to elucidate the role of fractionation regimen in the induction of the cognitive-sparing effect of FLASH-RT. They irradiated murine H454 Luc+ GBM tumors and U87 Luc+ human GBM cells after orthotopic injection of cells in the striatum of nude mice. For WBI, mice received 10 or 14 Gy as a single fraction; 4 × 3.5 Gy or 2 × 7 Gy daily fractionated WBI, or 3 × 10 Gy spaced by 48 h fractionated FLASH-RT or CONV-RT. Their data showed that FLASH-RT, delivered with a large neurotoxic single dose or hypo-fractionated regimens, did not elicit neurocognitive decrements in memory and learning in tumor-bearing mice [71]. Allen et al. investigated the therapeutic potential of hypofractionated FLASH-RT for the treatment of a human pediatric medulloblastoma brain tumor using a radiosensitive, 3-week-old juvenile female and male C57Bl/6 mice to evaluate adverse long-term neurological and cerebrovascular outcomes. Their data reported that hypofractionated WBI-FLASH-RT (2 × 10 Gy) ameliorated neurocognitive deficits induced by CONV-RT and maintained synaptic integrity and plasticity at the electrophysiological (long-term potentiation), structural (presynaptic Bassoon/post-synaptic Homer-1a proteins), and molecular (synaptophysin) levels in several brain regions [143]. In addition, the results pointed out that FLASH-RT benefits were associated with reduced neuroinflammation (activated microglia) and the preservation of the cerebral vascular structure, by minimizing microglia colocalized to vessels and maintaining aquaporin-4 expression levels [13,143].

Evaluations of neurocognitive outcomes have been constrained, even though clinical data indicate that this is the most significant area of worry. A limitation of object recognition assessments is that the dynamic range is limited. In a two-object test, the novelty preference varies between 60% and 67%, compared to a chance level of 50%, resulting in merely a 10% to 17% difference to identify variations. Moreover, the manner in which novel object recognition data are typically presented prevents visibility of the underlying data, which can pose a problem when responses vary. The Discrimination index is calculated by subtracting the time spent on a familiar object from the time spent on a novel object, then dividing this difference by the total time spent on both. An animal that attends to a novel object for 20 s and a familiar one for 10 s shows a 67% preference for novelty, but an animal that only focuses for 2 s on a novel object and 1 s on a familiar one also shows the same novelty preference, even though the two situations are distinctly different. To avoid this, a minimum duration of attendance can be set (e.g., 30 s). This removes distracted animals. An alternative method involves testing animals for extended durations, like 5 or 10 min, to gather as much attention time as feasible. Nonetheless, a significant portion of this additional time spent attending is devoid of information, as demonstrated by Clark and colleagues [144], who found that novelty preference decreases by 50% after 30–40 s. The preference for novelty begins at a low level, increases quickly, and then decreases steadily to a low point within 2–3 min. Clarke et al. [144] utilized a 30 s cutoff since, in their experience, novelty preference mostly dissipates after 60 s. The method used by Clark et al. has been demonstrated to prevent certain intrinsic limitations of these tests.

Aside from mice, the benefits of the FLASH-RT effect were reported in a dose-escalation trial in mini-pig and cat tumor patients, higher animal models that share more similarities in physiology and anatomy with human [15]. Vozenin and colleagues irradiated the skin on the back of a single mini-pig to various single doses from 22 to 34 Gy in 3 Gy increments, with either 6 MeV electron FLASH-RT at a dose rate of 300 Gy/s or CONV-RT at 0.083 Gy/s. Their data showed that, 48 weeks post-IR, FLASH-RT had been well-tolerated, with only mild skin depigmentation at the irradiated site. Additionally, FLASH-irradiated biopsies revealed a preservation of epidermal CD34+ stem cells in the hair follicle bulge, whereas the CONV-irradiated skin area displayed severe fibronecrotic lesions [15,32]. By way of extension, they performed a single dose-escalation FLASH-RT study (25–41 Gy) on six feline patients with locally advanced T2/T3N0M0 squamous cell carcinomas of the nasal planum. FLASH-RT led to observations of no fibronecrosis, no erythema, no inflammatory cell infiltrates, no wet desquamation, no hyperkeratosis, and no cutaneous remodeling. Furthermore, all six cat patients were shown to have an excellent functional outcome, with no impairment in their sense of smell and no major disturbance of food uptake [15].

Buonanno et al. showed that in normal human lung fibroblasts (IMR90), 4.5 MeV protons delivered at FLASH-RT dose rates of 100 Gy/s or 1000 Gy/s resulted in a decreased number of SA-galactosidase-positive cells 1 month after IR exposure [114]. In 2020, Fouillade et al. reported that FLASH-RT reduced DNA damage and lethality in normal human lung cells (MRC5 and IMR-90), spared lung progenitor cells from excessive damage, and limited the incidence of IR-induced senescence compared to CONV-RT [92]. Khan et al. exposed A549 lung adenocarcinoma, HT-29 colorectal adenocarcinoma, and MDA-MB-231 breast cancer cells spheroids to electron irradiation at FLASH-RT (90 Gy/s) or CONV-RT (0.075 Gy/s) dose rates. The results showed that the FLASH-RT sparing effect in spheroids was observed for doses of ≥10 Gy, and FLASH-RT led to a dose-modifying factor of around 1.3 for doses above 10 Gy [38].

On the other hand, it cannot be overlooked that there have also been many studies reporting no significant sparing effect following UHDRs [4,30,31,145,146]. For example, Smyth et al. conducted a dose-escalation study on a murine model using total and partial body (abdomen, head, and thorax) synchrotron irradiations at UHDRs of 37–41.3 Gy/s in the hope of calculating the equivalent doses of CONV-RT [4]. However, when examining TD_50_ data (the dose expected to cause toxicity in 50% of mice, such as severe diarrhea, moribund behavior, and significant body-weight loss), this research did not observe any FLASH-RT normal tissue-sparing effect [4]. Gaide et al. treated a patient with cutaneous T-cell lymphoma with a 15 Gy single electron dose delivered at a FLASH-RT dose rate of 166 Gy/s vs. a CONV-RT dose rate (0.08 Gy/s). They found that there was no difference for acute (erythema) and delayed (mild chronic dermatitis on skin biopsies) reactions between FLASH-RT and CONV-RT [147]. Moreover, if comparisons to conventionally produced beams were performed, generally not only dose rate, but also energy distribution and IR quality verified, making it complicated to attribute any differential impacts to the dose rate. For example, Doria et al. reported for the first time a quantitative in vitro study of dose-dependent RBE of 1–5 MeV laser-driven proton on Chinese Hamster lung fibroblast V79 cells in the UHDRs regime (>10^9^ Gy/s) as a single exposure (up to 5 Gy). A RBE of 1.4 ± 0.2 at 10% survival fraction, as compared to a 225 kVp X-ray source, was observed, comparable to previously published data on the CONV proton RBE [148].

### 6.2. Tumor Control

**Figure 3:**
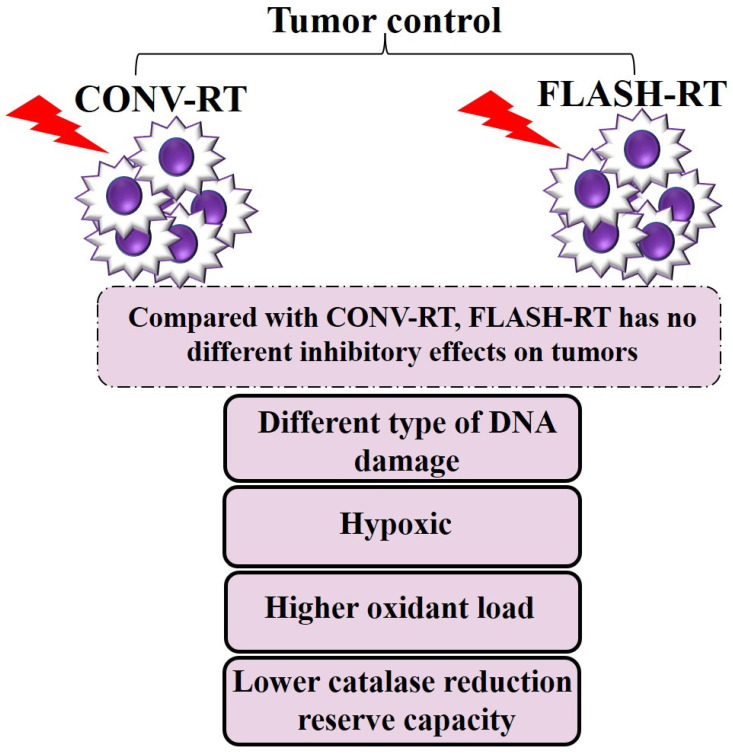
The anti-tumor efficacy of FLASH-RT vs. CONV-RT.

Several studies have reported that FLASH-RT achieves comparable tumor control efficacy to CONV-RT (Figure 3). The anti-tumor efficacy of FLASH-RT vs. CONV-RT has been shown in different mouse tumor models, such as orthotopic xenograft and transgenic models of head and neck, brain, breast, lung, and ovarian cancers [12] (summarized in Table A2). Levy et al. revealed that both 14 Gy TAI-FLASH-RT (216 Gy/s, electrons) and TAI-CONV-RT (0.079 Gy/s, electrons) exhibited a reduction in the total tumor weight and the number of tumor nodules in a murine model of metastatic ovarian cancer. Their findings suggested that large single fraction TAI-FLASH-RT may have great translational value if it holds in human patients [33]. Meanwhile, in preclinical lung tumor models, FLASH-RT and CONV-RT have been found to be equipotent in tumor control efficacy [149,150]. Favaudon et al. demonstrated that FLASH-RT (4.5 MeV electron) with a total dose of 15 Gy was efficient as CONV-RT (^137^Cs γ-rays) in the repression of syngeneic TC-1 Luc+ orthotopic lung tumors growth in C57BL/6J mice, whereas 23- to 28-Gy FLASH-RT doses were remarkably more efficient against the tumors [12]. Equal doses of FLASH-RT or CONV-RT were given to human HBCx-12A breast tumor, and human HEP2 head and neck carcinoma xenograft models. The observations also suggested that both treatment modalities achieved the same anti-tumor effect [12]. Montay-Gruel at al. reported that either hypofractionated or single-fraction WBI-FLASH-RT was equally capable as CONV-RT in delaying tumor growth in a murine orthotopic GBM model 1 month after irradiation [71].

The FLASH-RT effect on leukemia seems to be more subtle than its impact on solid tumor models. Chabi et al. used 6 MeV LINAC to investigate the effect of 4 Gy FLASH-RT vs. 4 Gy CONV-RT in T-cell acute lymphoblastic leukemia (T-ALL) patient-derived xenografts and healthy human hematopoietic stem/progenitor cells (HSPCs) and umbilical cord blood CD34+ cells transplanted in immunodeficient mice together or separately. In two of the three T-ALL xenograft models, TBI-FLASH-RT (200 Gy/s) was more efficient than TBI-CONV-RT (0.072 Gy/s) in controlling the tumor propagation, whereas the third case was more responsive to CONV-RT. In addition, TBI-FLASH-RT was able to maintain some CD34+/HSPC cell potential. When T-ALL and HSPC existed in the same animals, TBI-FLASH-RT exhibited a strong inhibitory effect on the growth potential of the tumor in three of four of the secondary grafted mice, whereas among the mice receiving TBI-CONV-RT, irradiated cells died due to high leukemic infiltration [131].

In the in vivo study by Bourhis et al. the striatum of nude mice was orthotopically inoculated with H454 Luc+ murine GBM cells. This was subsequently followed by WBI-FLASH-RT (1 pulse of 1.8 μs) or WBI-CONV-RT (0.1 Gy/s) 3 days post-implantation. The mice were exposed to a single fraction of 10 Gy, 3 × 8 Gy, or 5 × 5 Gy, with 24 h in-between fractions. The results indicated that there was no significant difference in tumor control between FLASH-RT and CONV-RT for any of the fractionation schemes [11]. Almeida and colleagues found that FLASH-RT and CONV-RT were equally effective in combating tumor growth in mice bearing SV2-OVA lung adenocarcinoma, and both modalities of irradiation were shown to increase the mean doubling time of tumors to >14 days post-IR compared to 8 days in nonirradiated controls [151].

Diffenderfer et al. found that both 12 and 18 Gy proton FLASH-RT did not exhibit tumor radioprotection in MH641905 pancreatic cancer flank tumors in the mouse model after TAI [39].

In some cases, the anti-tumor response to FLASH-RT might even be greater than that of CONV-RT. In a study by Rama et al. the left lung of C57Bl/6J mice was inoculated with non-small cell lung cancer (Lewis lung carcinoma). Two weeks after implantation, the whole lungs of tumor-bearing animals were exposed to a single dose of 18 Gy using a clinical pencil beam scanning at different dose rate modes of FLASH-RT and CONV-RT. Ten days post-treatment, the tumor sizes were measured with a caliper after the animals had been sacrificed. Surprisingly, the tumors of the mice treated with FLASH-RT were significantly smaller than those treated with CONV-RT [150]. In general, more recent in vivo studies investigating proton FLASH-RT have yielded much more positive outcomes by observing the anti-tumor effect of FLASH-RT compared to CONV-RT [27,36,96,119,120,121].

### 6.3. IR-Induced Immune Response in FLASH-RT

The contribution of the immune response has been thought to account for a specific fraction of the antitumor effectiveness of FLASH-RT. Multiple studies have shown that FLASH-RT contributes to the reduction in inflammation in normal tissues and the induction of an effective anti-tumor immunity, leading to better tumor control [115,152,153]. In addition, FLASH-RT has been shown to markedly decrease the fraction of circulating immune cells being irradiated and killed, resulting in a more potent immune system that can more efficiently repair IR-induced normal tissue injury [154,155]. Jin et al. reported that irradiation with a dose of ≥30 Gy in a single fraction decreased the circulating immune cells from 5 to 10% at extremely high dose rates of FLASH-RT to 90–100% at CONV-RT dose rates. Furthermore, they showed that the sparing effect on circulating immune cells decreased with reducing dose per fraction and with increasing the irradiated blood volume [65]. Girdhani et al. revealed that whole-thorax irradiation of C57BL/6 mice with single doses of 15, 17.5, and 20 Gy FLASH-RT (40 Gy/s, protons) reduced protein kinase C signaling in lymphocytes, dendritic cell maturation, T-helper 1 pathway modulation, and calcium-induced T lymphocyte apoptosis compared to CONV-RT (1 Gy/s, protons) [115]. Simmons et al. assessed the effect of WBI-FLASH-RT on neuroinflammation, factor associated with cognitive decline after brain irradiation. They administered 30 Gy WBI to C57BL6/J mice in 240 s CONV-RT vs. FLASH-RT (sub-second) delivery time. They evaluated expression of 10 pro-inflammatory cytokines (IL-1b, IL-2, IL-4, IL-5, IL-6, IL-10, IL-12, TNF-α, KC/GRO, and INF-γ), and neuroinflammation by CD68 immunostaining, a marker of activated microglia. They found that CONV-RT delivery time was associated with increased CD68-positive microglia 10 weeks post-IR, whereas FLASH-RT was not. Moreover, the results showed that FLASH-RT induced lower proinflammatory cytokine levels in the hippocampus 10 weeks post-IR, whereas CONV-RT delivery time was associated with significant elevations in levels of five of 10 pro-inflammatory cytokines [128]. Buonanno et al. investigated the expression of the pro-inflammatory marker TGF-β in IMR90 fibroblasts exposed in vitro to protons delivered at dose rates ranging from 0.05 Gy/s to 1000 Gy/s. Their results demonstrated that the expression of TGFβ1 was mitigated 24 h and 1 month after irradiation with 20 Gy of 4.5 MeV protons delivered at FLASH-RT dose rates [114].

Almeida et al. investigated the immune-modulatory effects of FLASH-RT in mice with various immune statuses using different fractionation regimens. They implanted murine different tumor models either orthotopically or subcutaneously into immunocompromised or immunocompetent mice. Subsequently, they exposed mice to single-fraction (20 Gy) or hypofractionated regimens (2 × 6 or 3 × 8 Gy) using CONV-RT (0.1 Gy/s) and FLASH-RT (≥2000 Gy/s) dose rates. Their data showed equipotency of immune profiles in SV2-OVA lung tumors after both irradiation modalities, with an increase in the proportion of myeloid cells and a reduction in the proportion of lymphoid cells by ~40%. In addition, their observations showed that intratumoral TGF-β1 expression levels were not elevated in SV2 OVA lung adenocarcinoma after both FLASH-RT and CONV-RT compared to nonirradiated control animals [151]. Furthermore, both irradiation modalities were able to generate long-lasting immunologic memory response against the GL261 GBM cell line [151]. The study by Rama et al. showed an improved recruitment of CD3+, CD+4, and CD+8 T cells from the peripheral tumor edge into the tumor core in the orthotopic Lewis lung tumor-bearing mice after irradiation with 18 Gy proton FLASH-RT at 40 Gy/s compared to proton CONV-RT [150].

Combination of FLASH-RT with immunotherapy, which is based on the functional execution of circulating immune cells, could be a proper strategy to boost anti-tumor immunity, potentially enhancing a patient’s quality of life.

### 6.4. Effects of FLASH-RT on the TME

The TME has been shown to be a major determinant for the therapeutic efficacy of RT. It has been reported that FLASH-RT may produce changes in the TME [156].

Kim et al. investigated the FLASH-RT effect on the TME in transplantable tumors grown in mice. They irradiated Lewis lung carcinoma, implanted subcutaneously in 6-week-old male C57BL/6 mice, with a single high dose of 15 Gy CONV-RT (0.06 Gy/s) or FLASH-RT (352.1 Gy/s) to determine the effect of FLASH-RT on the tumor vasculature. They observed contracted vessel morphology at 6 h after CONV-RT, whereas tumor vascular collapse did not occur with FLASH-RT. In addition, the in vitro observations suggested that myosin light chain kinase (ML-7) activation in Lewis lung carcinoma cells might be responsible for some of the microenvironmental alterations differentially regulated between FLASH-RT and CONV-RT. Furthermore, the results suggested that when combined with CONV-RT, an inhibitor of ML-7, it increased the CD31 area density, increased the γH2AX+ cells number, reduced the phosphorylated ML-7 expression, and increased the influx of immune cells (S100A8 myeloid cells and CD8α T cells) into the tumors, and these effects were similar to those observed in tumors irradiated with FLASH-RT in vivo [149].

## 7. The Most Popular Hypotheses on the FLASH-RT Effect Mechanism

Four competing, non-mutually exclusive hypotheses have been proposed to explain the potential advantages observed with FLASH-RT at biological and physicochemical levels: (1) oxygen depletion, (2) radical-radical interaction, (3) DNA damage, (4) and mitochondrial damage. Among those, the IR-introduced oxygen-depletion hypothesis has gained immense popularity [22,157].

### 7.1. Oxygen-Depletion Hypothesis

**Figure 4:**
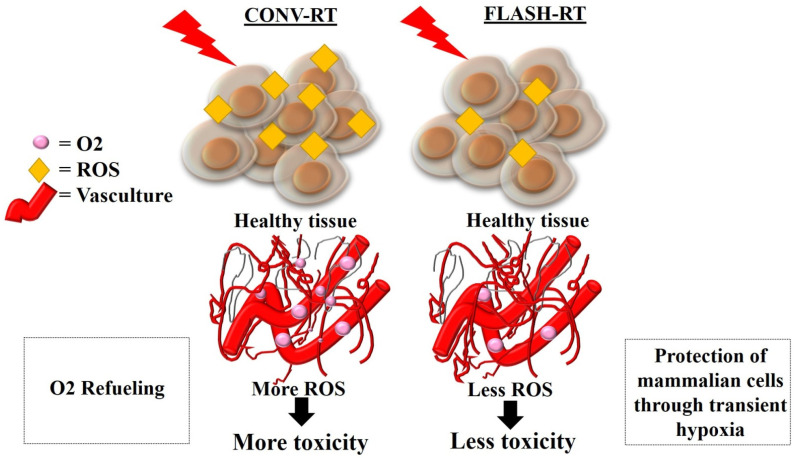
The FLASH-RT oxygen-depletion hypothesis. This phenomenon is not observed follow-ing CONV-RT. During CONV-RT, there is sufficient time for oxygen to diffuse back into normal cells and restore the oxygen that had been lost.

Many researchers have suggested that the differential response between CONV-RT and FLASH-RT is attributed to the rapid radiochemical oxygen depletion at UHDRs, and the subsequent radioresistance conferred to the IR-exposed normal tissue (Figure 4). In this short exposure time frame, the local oxygen-depletion process has been shown to be faster than diffusion and reoxygenation during FLASH-RT. Therefore, the normal tissue will be less radiosensitive than tumor tissue under FLASH-RT [4,50,154,158]. It is widely accepted that hypoxic cells are approximately three times less radiosensitive than well-oxygenated cells [159]. When considering the oxygen-depletion hypothesis, the concentration of dissolved oxygen within the tissues and oxygen partial pressure (pO2), also known as “physioxia” or “physiological normoxia”, must be accounted for [160]. Oxygenation measurements in normal tissues have shown that they display distinct normal ranges, which vary between tissue types. It has been reported that physioxia generally ranges from 3.4 to 6.8% oxygen, with an average of around 6.1% [161]. Depending on the tissue of origin, the median oxygen levels in untreated tumors have been shown to lie between 0.3% and 4.2%, with most tumors displaying median oxygenation levels, 2% [161].

Multiple in vitro studies have indicated that after a certain FLASH-RT dose, cells begin to behave as if they were hypoxic [158,162]. FLASH-RT has been shown to transiently consume the local oxygen in tissues via the following reactions: e_aq_^−^ + O_2_ → O_2_^•−^, H^•^ + O_2_ → HO_2_^•^, producing a significant decrease in oxygen level, thereby preventing DNA from transitioning into an irreversibly damaged state [47,163]. On the other hand, CONV-RT has been shown to involve the delivery of doses in a timescale of minutes, which is much longer than the rediffusion of oxygen time through tissue, resulting in only a minor decline in oxygen content [164].

Healthy tissue cells have been found to have a greater catalase-reduction reserve capacity and lower oxidant loads; thus, healthy cells can efficiently remove these products than tumor cells [165]. A relationship between the IR dose rate and the oxygen content was first put forward in 1959 by Dewey and Boag [8]. They irradiated *Serratia marcescens* using UHDR MV X-rays. The study demonstrated that bacteria irradiated at UHDRs (100–200 Gy/2 μs) had lower radiosensitivity in a gas mixture containing 1% oxygen and 99% nitrogen compared to those exposed to what we now consider to be CONV-RT dose rates (10 Gy/min) in 100% nitrogen, and this lower radiosensitivity corresponded to anaerobic irradiations. The authors attributed this response to the radiolytic removal of dissolved molecular oxygen from the interior of the bacterium; the time for which these bacteria were irradiated was much shorter than the time needed for oxygen replenishment through diffusion [8,166].

The oxygen-depletion theory has been reinforced by studies exhibiting that clonogenic survival of cells following UHDR irradiation mimics that of cells irradiated in hypoxic conditions [167]. An in vitro study by Michaels et al. used single electron pulses of 3 ns duration and a UHDR of 10^9^ Gy/s to irradiate Chinese hamster ovary cells at relatively low oxygen concentrations (0.44–0.7%). Their results showed that the breaking behavior of the survival curve when cells were equilibrated with 0.44% oxygen was consistent with a radiolytic oxygen-depletion process induced by the IR pulse, and the breakpoint dose was found to be approximately linear with respect to increasing the oxygen concentration [137]. Furthermore, previous publications have shown that FLASH-RT is most effective when applied to hypoxic cells/tissues. For example, Adrian et al. compared FLASH-RT (600 Gy/s) to CONV-RT (14 Gy/min) at doses ranging from 0 to 25 Gy under various oxygen levels (relative pO2 ranging between 1.6% and 20%). The in vitro results showed a significant increase in the survival of hypoxic DU145 prostate cancer cells (1.6% oxygen concentration) when exposed to FLASH-RT at 18 Gy compared to CONV-RT, whereas no differences in the IR response between the two modalities under normoxic conditions were found [50]. Another in vitro study revealed that the proportion of early apoptosis, late apoptosis, and necrosis were obviously lower in FLASH-RT-irradiated normal mouse embryonic fibroblast cells in hypoxia than that of normoxia, indicating that resistance of these cells to FLASH-RT can be increased by hypoxia [168]. Hendry et al. revealed that the pulsed electron beam irradiation of mouse tails with a pulse duration of <4.5 s in air induced epithelial resistance to necrosis, indicative of oxygen depletion [37]. Taylor et al. investigated the impact of spatial heterogeneity of the pO2 on the differential effect of FLASH-RT on tumor response and normal tissue toxicity by simulating oxygen diffusion over domains containing oxygen sources (capillary architectures). Their results showed that the presence of poorly oxygenated regions in well-perfused normal tissues with high mean pO2 led to greater proportional normal tissue sparing than tumor cells during FLASH-RT. In addition, their findings showed that in the presence of anoxic (i.e., pO2 exactly zero) regions in well-oxygenated (normal) tissues, FLASH-RT could not further deplete oxygen from tissues and the survival curves for CONV-RT and FLASH-RT were the same [51]. Khan et al. used 3D multicellular tumor spheroids as in vitro models to investigate FLASH-RT-induced depletion of oxygen. A549 lung adenocarcinoma spheroids irradiated to a 16 MeV electron beam at 10 Gy FLASH-RT (90 Gy/s) showed a transient increase in hypoxia in comparison to the CONV-RT dose rate (0.075 Gy/s) [38].

Most tumors have been shown to be not completely anoxic [161]. Hence, FLASH-RT will also lead to oxygen depletion within the tumor. It is possible that this would confer radioresistance to the tumor and thus, would reduce tumor control [169]. Nevertheless, minimal reductions in tumor control would likely be seen in highly hypoxic tumors. Cao et al. measured the changes in oxygen per unit dose (G-values) after irradiation with a 10 MeV electron beam at either a CONV-RT dose rate of 0.1 Gy/s or a FLASH-RT of 300 Gy/s. Their in vitro experiments in aqueous solutions (containing 5% bovine serum albumen) led to a G-value for radiochemical oxygen depletion of 0.16 to 0.17 mm Hg/Gy for FLASH-RT and a 0.19 to 0.21 mm Hg/Gy for CONV-RT [164]. Additional experiments using the MDA-MB-231 mouse model showed that the total decrease in oxygen following a single fraction of 20 Gy FLASH-RT was 1.0 mm Hg in tumor tissue and 2.3 mm Hg in intact tissue, whereas no reduction in the availability of oxygen was observed upon application of CONV-RT [164].

### 7.2. The Radical-Radical Interaction

The free radical interaction hypothesis has suggested that UHDR of FLASH-RT leads to instantaneous, transient accumulation of a large concentration of organic free radicals R^•^ and organic peroxyl radicals ROO^•^ to react with each other and form stable, non-radical adduct [170]. The increased probability of radical recombination under FLASH-RT has been reported to shorten the redox chain reactions, subsequently resulting in less oxidation damage to healthy tissue. This includes reducing protein and lipid peroxidation as well as alleviating DNA damage. Meanwhile, other free radicals can react with oxygen to produce ROS [171,172,173]. It has been believed that tumor cells exhibit elevated steady-state fluxes of ROS (i.e., O_2_^•−^, H_2_O_2_, and HO^•^) and redox active metal ions (i.e., labile iron, also known as protein-bound iron or non-free iron (Fe^2+^)) due to defects in oxidative metabolism [165,174,175].

Spitz et al. [165] presented a series of reasonable explanations for the maintained tumor control under FLASH-RT: (1) FLASH-RT doses that consume all locally available oxygen in tissue to form reactive organic hydroperoxide products will display the maximal effective differences between normal and tumor tissues; (2) normal cells, relative to tumor cells, have two- to four-fold less levels of labile iron (released from iron-containing proteins, i.e., ferritin, aconitase, and Fe-S proteins by interacting with O_2_^•−^, which is generated from O_2_ reacting with aqueous electrons e_aq_^−^ and H^•^ generated from the interaction in water during the FLASH-RT), accompanied by decreased levels of transferrin receptor; (3) normal cells have a larger ability to sequester labile iron pool compared to tumor cells; hence, they are less susceptible to Fenton-type chain reaction (Fe^2+^ + H_2_O_2_ → Fe^3+^ + OH^•^ + OH^−^) that is known to magnify oxidative damage to macromolecules; (4) normal cells also have lower prooxidant burdens during normal steady-state metabolism and a greater reserve capacity for the enzymatic reduction in hydroperoxides, relative to tumor cells.

Radiochemical studies by Montay-Gruel et al. showed that FLASH-RT produced significantly less hydrogen peroxide in aqueous solutions containing 4% oxygen at all doses above 10 Gy (30, 40, 50, 60, 70, and 80 Gy) compared to CONV-RT [24]. Notwithstanding, H_2_O_2_ cannot fully represent ROS, and because of the differences between in vitro and in vivo conditions, their mechanisms could differ [176]. Scavenging studies implementing millimolar concentrations of two well-known ROS scavengers (N-acetyl-cysteine, and amifostine) were shown to have no impact on body length of FLASH-irradiated zebrafish embryos. However, zebrafish embryos exposed to CONV-RT alone were significantly shorter than those preincubated with either ROS scavenger [24].

### 7.3. DNA Damage

Among the discovered factors, DNA damage, especially unrepaired DNA-double strand break (DSB), has been shown to be an intrinsic and primary factor, and the most essential operator in the response to IR-exposure [177,178,179,180]. It has been suggested that a differential response to FLASH-RT between tumor and normal tissues results from a combination of two intermingled mechanisms pertaining to the formation of IR-induced DNA damage and its repair [173]. (1) DNA damage yields: endpoints including intercellular distribution of micronuclei, chromosome aberrations (mainly dicentrics), and G2 arrest have suggested that FLASH-RT generates less DNA damage than does CONV-RT in normal cells [181,182]. A significant decrease of DSB-induced γH2AX foci was observed in IMR90 cells 30 min after exposure to 20 Gy of 4.5 MeV protons delivered with FLASH-RT (1000 Gy/s) when compared to CONV-RT dose rates [114]. Cooper et al. assessed DNA damage levels (% Tail DNA) in healthy human peripheral blood lymphocytes in vitro after exposure to a 6 MeV electron beam at either FLASH-RT (2000 Gy/s) or CONV-RT dose rates of 0.1 Gy/s at different oxygen levels (0.25–21%), with different total doses (5–40 Gy) and intermediate dose rates (0.3–1000 Gy/s) [183]. Their findings revealed that different factors, such as oxygen tension, total dose, and dose rate, influence the amount of DNA damage caused by FLASH-RT. Moreover, they showed that FLASH-RT induced lower levels of DNA damage at dose rates of ≥30 Gy/s and total doses of ≥20 Gy, at 0.5% oxygen [183]. FLASH-RT dose rates have been found to contribute to an increase in clustered DNA damage [182,184,185]. (2) DNA damage pattern: high LET particles have been shown to induce more clustered DNA damage than regular photons. Most FLASH-RT preclinical experiments have used electrons and photons, and a few studies have used protons [186]. The LET of these irradiations has been found to be insufficient to cause clustered DNA lesions if they are delivered at the CONV-RT dose rate; however, if they are conducted at UHDR, the DNA damage pattern is not expected to be very different [11,98]. Of importance is that low-energy (high LET) protons are thought to produce complex DNA damage with high frequency, whereas high-energy (low-LET) protons generate DNA lesions similar to that of γ- and X-irradiations. The difference between tumor cells and normal cells in response to clustered DNA damage possibly results in the activation of different factors (DNA repair or immune system) [187]. (3) DNA damage repair pathways: in normal cells, there are many highly conserved DNA repair pathways, and the activation of each pathway varies depending on the cell type and the cell-division cycle status [188,189]. Non-homologous end joining (NHEJ) has been reported to be dominant throughout the whole cell cycle, whereas homologous recombination (HR) functions primarily in the late S/G2 phases of the cell cycle [190,191]. Disruptions in DNA repair pathways or cell cycle checkpoints have been shown to cause changes in the cellular response to IR [192,193,194]. Several reports have pointed to the widespread involvement of Poly (ADP-ribose) polymerases (PARPs), chromatin-bound enzymes, in various cellular functions, including regulating chromatin structure, replication, transcription, DNA repair response, and mammalian innate immune response involved in pathogenic infections, inflammations, and tumors [25]. PARP-1, -2, and -3 catalytic activity has been shown to be stimulated by and promote the repair of DNA single-strand breaks (SSBs). PARP-1, the most abundant isoform, has been shown to be responsible for more than 90% of PARP functions in both normal and transformed cells [195,196]. PARP-1 has been shown to control the duration and strength of SMAD-mediated transcription and regulate SMAD-dependent responses to TGF-β, such as epithelial-mesenchymal transition (EMT) [197,198]. Inhibition of PARP family members have been shown to result in the accumulation of DSBs [196]. It is noteworthy to mention that the majority of human cancers have mutations in DDR signaling pathway genes such as CHK1 and CHK2, BRCA1 and BRCA2, MSH1 and MSH2, and RAD51C and RAD51D, which are DNA damage checkpoint genes, mismatch repair, DSBs repair, and homologous recombination, respectively [199,200,201,202,203,204,205]. (4) DNA damage-induced factors: Many studies have revealed that IR-induced double-stranded DNA (dsDNA) might become cytosolic dsDNA, which activates the cyclic cytosolic DNA sensor GMP-AMP synthase (cGAS) and its downstream effector, interferon (IFN) genes (STING). The cGAS–STING signaling pathway has been known as a key component of the innate immune system that recognizes cytosolic dsDNA as a danger-associated molecular pattern, contributing to the production of type I IFNs and other innate immune inflammatory factors that can lead to cellular senescence, autophagy, cell death, or tissue injury. In addition, the cGAS–STING pathway, when activated, has been shown to boost the immunogenicity of tumors and the activity of dendritic cells [199,200,201,202,203]. This pathway in different tumor cells depends on several factors, involving tumor cell type, stage, and tumor immune microenvironment [206]. Altered regulation of the secretion of inflammatory chemokines and cytokines after CONV-RT has been shown to promote IR-induced lung fibrosis [207]. Differential cGAS-STING pathway activity between normal and tumor cells could result in the FLASH-RT effect, potentially impeding tumor growth while protecting normal tissue from severe injuries, such as pulmonary fibrosis [203].

### 7.4. Mitochondrial Damage

**Figure 5:**
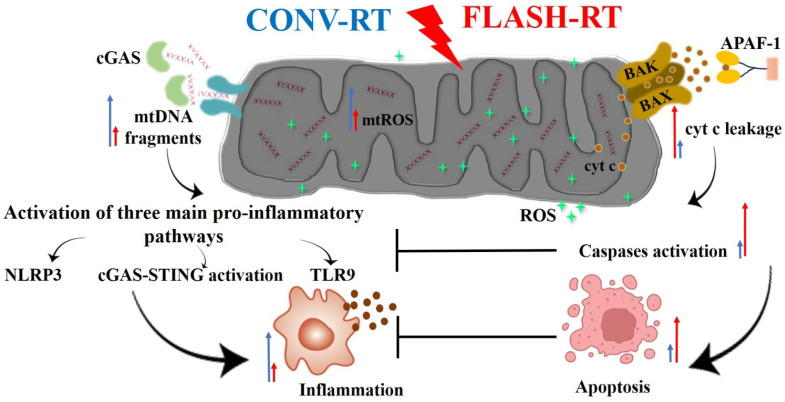
FLASH-RT may cause marginal mitochondrial damage, resulting in less apoptotic death and less inflammation compared to CONV-RT. IR has been shown to cause mtDNA damage either directly by interacting with it or indirectly through ROS generated by the ETC. Mitochondrial dysfunction is usually accompanied by elevated endogenous ROS levels. Mitochondrial dysfunction has been shown to activate pro-apoptotic proteins BAK and BAX, which further elicit mitochondrial outer membrane permeabilization (MOMP), leading to the release of cyt c into the cytosol, thus initiating apoptotic signaling cascades. MOMP has been found to facilitate the release of mitochondrial-derived damage-associated molecular patterns, such as oxidized mtDNA fragments, via mPTP, triggering the inflammatory response through the activation of various pro-inflammatory signaling pathways: TLR9, cGAS–STING/TBK1, and NLRP3. Abbreviations: BAX, BCL-associated X; BAK, Bcl2 homologous antagonist/killer; cyt c, cytochrome c; APAF-1, apoptotic protease activating factor-1; cGAS–STING, GMP-AMP synthase–stimulator of interferon genes DNA-sensing system; mPTP, mitochondrial permeability transition pore; TLR9, toll-like receptor 9; NLRP3, nucleotide-binding oligomerization domain-like receptor family pyrin domain containing 3.

The core idea of this interesting hypothesis is that mitochondria-mediated apoptotic and inflammatory responses might be significantly decreased following FLASH RT compared to CONV-RT. Mitochondria have been found to be highly engaged in cell signaling, Ca^2+^ homeostasis, redox metabolism, and apoptosis [208]. Furthermore, they have been found to play a pivotal role in regulating the innate immune system and inflammatory pathways [209]. Mitochondria have also been shown to be the primary producers of cellular ROS (mtROS), which act as a stimulating agent for cell proliferation and survival under physiological conditions. However, mtROS overproduction in malignant cells has been shown to cause oxidative damage that affects many cellular components, such as proteins, lipids, and mitochondrial DNA (mtDNA) under cellular stress, thereby triggering cell dysfunction and/or death. Even small elevations in mtROS might give rise to a critical excess of ROS in tumor cells [210]. This may possibly explain why a similar anti-tumor effect is observed after FLASH-RT vs. CONV-RT. It has been believed that FLASH-RT can minimize mitochondrial damage and/or mtROS production in normal cells compared to CONV-RT (Figure 5) [152]. Therefore, many researchers are curious about the potential role of mitochondria in the FLASH-RT effect and the impact of this cutting-edge technology on their function.

The effect of FLASH-RT on mitochondrial function was explored in IMR90 fibroblasts that were irradiated to 15 Gy protons (LET = 10 keV/µm) delivered at either CONV-RT (0.33 Gy/s) or FLASH-RT (100 Gy/s) under an ambient oxygen concentration (21%). Compared to CONV-RT, FLASH-RT resulted in a dramatic reduction of mitochondria damage, characterized by morphological changes, functional changes (mitochondrial copy number, membrane potential, and cellular ATP levels) and IR-induced oxidative stress. In contrast, both of the IR modalities negatively influenced mitochondrial morphology and cell viability of A549 lung cancer cells. Based on these data, Guo et al. proposed that FLASH-RT preserved mitochondria function of normal lung fibroblasts but not cancer cells through the induction of the phosphorylated form of dynamin-1-like protein (p-Drp1), while CONV-RT led to dephosphorylation of the p-Drp1 and induced Drp1 to be aggregated into mitochondria, which ultimately caused mitochondria fission and cell necrosis [211]. Han et al. carried out a study on this topic, employing FLASH-RT (~10^9^ Gy/s ultrafast laser-generated particles) to irradiate normal cytochrome c-defective (cyt c−/−) and (cyt c+/+) mouse embryonic fibroblast cells at the dose of ~10–40 Gy under hypoxia-like conditions mimicked by CoCl_2_ addition and normoxic conditions. In both hypoxic and normoxic conditions, FLASH-RT-irradiated cyt c−/− cells showed lower levels of late apoptosis and necrosis compared to cyt c+/+ cells, indicating that the loss of cyt c may enhance the protection of normal fibroblast cells during FLASH-RT [168].

Apoptotic caspases have been shown to be capable of inhibiting mitochondria-dependent inflammatory response during cell death [212]. To further explore the role of these organelles in the FLASH-RT effect, Lv et al. observed that compared to the electron CONV-RT dose rate (0.36 Gy/s), FLASH-RT (61 or 610 Gy/s) triggered cyt c release from mitochondria into the cytosol in normal human breast cells MCF-10A [213]. This result suggested that FLASH-RT may activate cyt c-caspases chain-mediated apoptosis and suppress the IFN-β activation mediated by the cytosolic mtDNA-induced cGAS-STING signaling axis, thereby minimizing the immune damage to non-targeted tissues post-IR [213]. Intriguingly, the findings in carcinoma cells MDA-MB-231 following FLASH-RT were completely the opposite, namely, less cyt c release and less caspase activation but stronger cGAS-STING signaling activation than those in MCF-10A cells [213]. The difference of cyt c release between normal cells and cancer cells post-FLASH-RT indicated a potential mechanism of FLASH-RT effect by orchestrating the mitochondria-mediated apoptosis and inflammatory response [213]. Besides, the authors [213] explained this difference by the “Warburg effect” [214] that most tumor cells tendentiously release energy through anaerobic glycolysis in the cytosol instead of the usual citric acid cycle and oxidative phosphorylation in the mitochondria, even in the abundant presence of oxygen. Furthermore, Lv et al. reported that FLASH-RT did not promote significant morphological alterations in mitochondria in MCF-10A cells, but it increased mitochondrial fission in MDA-MB-231 cells. The authors also reported that the variation of cyt c leakage with UHDR was inconsistent with the morphological changes of mitochondria [213]. Based on these results, it was proposed that FLASH-RT promotes an extensive cascade feedback of electron transport chain (ETC) dysfunction, dissociation, and leakage of cyt c from the inner mitochondrial membrane in normal cells [213].

The reduced mitochondrial damage in vivo could be due to the potential protective effect of circulating immune cells at FLASH-RT dose rates [215]. Regarding the effects of FLASH-RT, the mitochondrial response has not yet been thoroughly investigated. Comparable studies on mtROS/mtDNA leakage following FLASH-RT in both tumor and normal cells will be crucial to offer convincing evidence.

## 8. The Translational Potential of FLASH-RT to the Clinical Environment

Many studies have championed the use of FLASH-RT. FLASH-RT strategies could be transferred into clinical trials to serve two main purposes: Firstly, FLASH-RT radiobiological advantages could be exploited to allow for safe and possibly efficacious total dose escalation in the treatment of radioresistant tumors that are related to poorer satisfaction and outcomes of patients [11]. In this case, it has been suggested that a higher total dose could be administered to the tumor without causing as severe damage to nearby healthy tissues as would be expected following CONV-RT treatment. Secondly, FLASH-RT could be applied in situations in which RT confers good tumor control rates but is associated with unacceptable levels of normal tissue toxicities—the same total dose would be delivered, but FLASH-RT would reduce potential adverse side effects in normal tissues compared to CONV-RT. Although FLASH-RT shows promise for clinical applications, its suitability for actual clinical practice is uncertain. There are some discrepancies in the results of preclinical trials [4,146]. Moreover, some of the FLASH-RT clinical trials have been found to have deficiencies, such as the absence of a CONV-RT control group and the use of individual subjects [15,184].

In 2019, Bourhis et al. reported the first FLASH-RT human treatment of a patient with a multiresistant CD30+ T-cell cutaneous lymphoma disseminated over the whole skin at Lausanne University Hospital in Switzerland [2]. In the first FLASH-RT of a human subject, an average dose rate of 167 Gy/s was delivered for treatment of a 3.5-cm-diameter skin tumor with a 5.6 MeV LINAC and a prescribed dose of 15 Gy to the planning target volume was selected, in 90 ms. The researchers observed a noteworthy sparing effect of FLASH-RT in their study. Furthermore, tumor shrinkage was observed 10 days post-FLASH-RT, culminating in a complete tumor response 36 days post-FLASH-RT, which was maintained for the next 5 months. From the point at which the tumor began to shrink, the patient presented with redness and a transient grade 1 oedema along with grade 1 epithelitis in the skin surrounding the tumor. This was starkly different for the patient with various cutaneous lesions subjected to 20 Gy CONV-RT as 10 fractions, or 21 Gy in six fractions, that led to poor skin tolerance despite the relatively low RT total doses used, with severe acute skin reactions that took 3–4 months to heal for a 3–4 cm diameter lesion [2]. In light of these encouraging results, it is important to note that this does not conclusively prove that FLASH-RT can be effectively implemented in clinical practice. This trial was conducted with only a single patient, which restricted the ability to fully compare the differential responses between FLASH-RT and CONV-RT. A properly randomized controlled trial with FLASH-RT and CONV-RT arms would be required to definitively demonstrate whether FLASH-RT results in improved clinical outcomes. At the least, a positive phase II single-arm clinical trial of FLASH-RT in a truly representative sample of real-world patients is needed before the application of FLASH-RT can be seriously entertained.

In 2022, Bley et al. published the first paper that highlighted several caveats related to the translational potential of FLASH-RT to the clinic, and demonstrated that application of a very high single dose of irradiation and a large field size of surface exposure will present formidable difficulties for minimizing late normal tissue toxicities [21]. In their study, feline patients with T1/2, N0 carcinomas of the nasal planum were randomly allocated to two arms of electron-beam RT: standard-of-care treatment (arm 1) was implemented using 48 Gy at a dose rate of 6 Gy/min; arm 2 received 30 Gy FLASH-RT at an average dose rate of 1500 Gy/s. Mini-pigs were treated using applicators of enhancing field size and a single dose of 31 Gy FLASH-RT delivered in 10 pulses of 1.8 ms [21]. Cats experienced mild and similar short-term side effects in both arms. Three of seven cats (43%) developed osteonecrosis of the maxilla at 9 to 15 months post-FLASH-RT, compared to 0 of nine cats treated with the standard of care. In FLASH-RT–treated pigs, no short-term toxicity was observed, but severe long-term cutaneous necrosis occurred 7 to 9 months later. All cats in both groups were free of progression at 1-year post-treatment [21].

The first-in-human FAST-01 non-randomized trial (ClinicalTrials.gov identifier: NCT04592887) evaluated the clinical workflow feasibility of proton FLASH-RT in the treatment of symptomatic bone metastases in the extremities for a small number of people. The FAST-01 findings were published in the *Journal of the American Medical Association Oncology* on 23 October 2022 [36,70]. In 2023, the FAST-02 trial, designed to test proton FLASH-RT for the treatment of bone metastases in the chest, was opened for enrollment [29]. Furthermore, the current evaluation of FLASH-RT in clinical settings has been shown to be restricted due to the lack of appropriate IR delivery systems. Although current systems can be adapted to provide FLASH-RT, a novel generation of machines will be necessary for this technology to be more widely available in clinical settings [19,43,216,217]. Small-animal RT devices are progressively sophisticated but remain incapable of recapitulating medical LINACs in terms of dose rate and beam energy [218,219]. The use of VHEE has been demonstrated to be limited due to technical issues related to electron acceleration in a conveniently-sized medical device (i.e., a device that is neither too big nor too complicated) [23]. The pluridirectional high-energy agile scanning electron RT (PHASER) might be an optimal way for clinical translation of FLASH-RT. This platform might enable RT delivery quickly enough to freeze the tumors’ motion, solve the problems of size and stability of VHEE beams, and also allow for the generation of 6–10 MV FLASH X-ray beams [23]. Involved in this concept is a cutting-edge and quick image-guided technique. Certainly, highly adapted image-guidance technologies are necessary for delivering FLASH-RT dose rates to deep-seated tumors, regardless of IR modality.

## 9. Challenges Related to Clinical Translation of FLASH-RT

**Figure 6:**
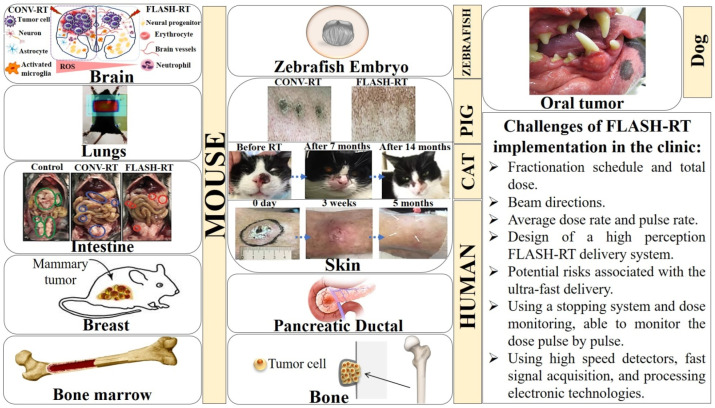
FLASH-RT experiments and limitations in clinical trials: Studies in animals (mice, pigs, cats, zebrafish, and dogs) have led to further development in FLASH-RT. However, there remain various difficulties in studying its application in humans, in terms of dosage, equipment, IR source, and economical factors.

Improved optimization of parameters and technological hurdles are essential for enabling the clinical implementation of FLASH-RT. There are great opportunities, but also many challenges for the high-energy and particle-physics communities to produce the desired dose rates in FLASH-RT (Figure 6). These challenges differ significantly depending on the type of accelerator used to generate the relevant UHDR-IR environment. There is a lack of suitable large-area monitors and satisfactory in vivo dose-monitoring systems for FLASH-RT [34]. In addition, it is essential to rely on dosimeters that are proven to function accurately in extreme IR conditions, i.e., passive dosimeters. Motion accounting is a prerequisite for FLASH-RT. It has been demonstrated that respiratory motion complicates RT, leading to the inclusion of the concept of “time” as a key factor in RT [14,220]. Despite the generic notion that the FLASH-RT delivery is instantaneous and minimizes the motional influence, the interplay effect between internal motion and UHDR delivery is one of the still outstanding great challenges of FLASH-RT. A sudden temporary motion in FLASH-RT means that the total dose is administered incorrectly [29]. This concern about rapid timing underscores the importance of correctly identifying motion states and visualizing oxygen distributions to guarantee precise and safe delivery. Current state-of-the-art dosimeters do not have the needed temporal resolution to deliver the UHDRs of FLASH-RT. Motion management for UHDR delivery needs development by improving ultrafast and pulse-based in vivo dosimetry. Furthermore, respiratory gating techniques can be employed by ensuring consistent target positioning, monitoring residual target motion, and activating the beam within the gating window. Before participating in clinical trials, these motion-management tools must be certified via a comprehensive moving phantom test. Detectors such as diodes, ionization chambers, and diamond detectors reach saturation when the dose per pulse/dose rate exceeds CONV-RT dose rates [221,222], which necessitates the use of beam-quality correction factors. The constraints of delivering FLASH-RT pose challenges across different areas of RT technology. Although electron beams are commonly utilized in preclinical FLASH-RT research, they are not effective for treating deep-seated and large target volumes and have limitations in achieving sufficient conformity with the field dose [223], restricting their use in delivering FLASH-RT to different disease sites. Additionally, there are significant technical challenges in generating X-ray FLASH-RT beams within the megavoltage energy range, limiting the use of photon FLASH-RT. Moreover, present scientists are investigating various methods, including redesigning X-ray tubes [224] and enhancing the design of LINAC targets [23], in order to produce X-rays FLASH-RT. These research projects are still in the early stages, so more investigation and progress are necessary before any practical implementation can be done. Proton FLASH-RT is not commonly accessible because of its costly infrastructure and increased operating expenses. It requires a significant financial commitment for acquiring specialized equipment. Substantial advancements are needed to facilitate proton FLASH-RT delivery, especially if the goal is to modify current proton spot-scanning systems for treatment at FLASH-RT dose rates in clinically significant areas. The lack of commercially available treatment planning systems for dosimetry studies that can accurately incorporate and simulate the distinctive features of FLASH-RT complicates the development and validation of proton FLASH-RT methods. Researchers have been searching for innovative solutions to overcome these challenges and provide broader access to FLASH-RT. One pursuit is to develop a single-energy Bragg peak FLASH-RT technique for conformal RT, eliminating the need for energy selection and transportation systems, ultimately reducing the size of proton systems. The use of protons at a single energy level in the single-energy Bragg peak FLASH-RT approach simplifies the UHDR delivery to achieve intensity-modulated proton therapy-like dosimetry quality through multiple-field delivery and eliminates the exit dose linked to transmission proton FLASH-RT, all while better protecting organs at risk. Additionally, this strategy can reduce technological barriers and expenses, opening up new prospects for affordable FLASH-RT [118,225]. Geographical differences in the availability of proton therapy [225] need further investigation as they could expand access to FLASH-RT for more patients.

Before FLASH-RT can be established as the primary RT technology in clinical use, additional animal studies are necessary. The delivery needs to be adjusted for broader fields of RT, include conformal IR across multiple areas, reevaluate dose limits for healthy tissue, and apply radical irradiation doses for tumors.

## 10. Conclusions

At the present time, no RT method is perfect. The major drawback of RT treatment is that, in order to deliver a lethal dose to tumor cells, short- and long-term side effects are obvious due to the irradiation of the surrounding healthy tissues that can seriously affect the quality of life of tumor patients. The rapid delivery of high doses of IR in a short time, known as FLASH-RT, is being recognized as a promising treatment approach to enhance the differential effect between normal tissues and tumors. For FLASH-RT, the experimental animal models (e.g., rodents, zebrafish, mini-pigs, cats, dogs) are, and will remain, vitally important for radiation oncology clinical research. There has been a lot of speculation about the biological mechanisms behind the FLASH-RT effect. From the preclinical data currently available, it can be safely inferred that radiolytic oxygen consumption contributes, at least partially, to the FLASH-RT effect. However, the extent of its contribution merits further investigation. Besides the inherent differences in ROS and free radicals’ generation between normal and tumor tissues, DNA integrity, mitochondrial homeostasis, and immune modulatory roles have been widely included in the FLASH-RT effect; yet evidence to substantiate this is currently preliminary. Many unresolved questions remain, including the specific conditions in which healthy tissue preservation can be observed. From a practical standpoint, FLASH-RT can be particularly beneficial for treating stubborn tumors located in the brain, gastrointestinal region, or lungs, as the healthy tissues around these tumors are especially sensitive to IR. Nonetheless, clinical trials at these areas cannot be approved until research demonstrates that UHDR-IR is both safe and effective in different, less-radiosensitive sites. Treatment of extremity bone metastases involves areas at a significantly lower risk for complications than when treating the brain or lungs. Furthermore, findings from FLASH-RT tests performed on radiosensitive juvenile animals indicate the encouraging possibility of applying FLASH-RT to deliver much-needed relief for children scheduled for RT targeting medulloblastoma, the most prevalent malignant brain tumor in children. Consequently, FLASH-RT may also aid in the treatment of pediatric tumors, given that children are generally more susceptible to the side effects of RT. However, significantly more research must be carried out before that can occur. The effects of electron FLASH-RT exposure can be applicable in mice since these animals are diminutive, allowing electrons to penetrate their brains adequately to trigger the FLASH-RT effect; however, it remains uncertain if the data serve as a reliable translational model. Like any pioneering research, challenges with funding and procedures still exist. The significant costs and limited availability of FLASH-RT are further exacerbated by its heightened technological and operational requirements. Furthermore, there are difficulties in applying FLASH-RT in clinical settings, especially in ensuring accurate dose distributions and treating tumors located deep within the body using electron beam therapy. Although experiments at FLASH-RT dose rates using protons have been conducted, they have only been performed on small volumes up to now. Dosimetric investigations are crucial for accurately measuring and monitoring the IR doses administered in treatment to guarantee efficacy, safety, and precision of RT. Researchers face significant obstacles in accurately ascertaining treatment parameters and refining treatment schedules due to the absence of specialized dosimetry tools for FLASH-RT. There is an increasing need for vendors to incorporate novel FLASH-RT treatment planning methods into treatment planning systems. While CONV-proton beam therapy results in high doses being delivered to surrounding organs or healthy tissues outside of the target area, a new Bragg peak FLASH-RT technique has been created to provide precise doses with single-energy proton beams, capitalizing on the benefits of proton therapy. It is believed that integrating both ridge filter and single-energy Bragg peak FLASH-RT techniques into treatment planning systems is a major step forward for conformal FLASH-RT techniques. The ridge filter enables the adjustment of Bragg peak broadening based on the need for dose shaping in the target volume, whether for a spot or energy layer. It greatly decreases the number of energy layers and spots needed to cover the target volume, leading to a reduction in treatment time. Using the single-energy Bragg peak FLASH-RT method maximizes the advantages of the Bragg peak for UHDR therapy and provides a more cost-effective solution by reducing the need for intricate beam modulation in implementing FLASH-RT. Incorporating these methods into treatment planning platforms would allow for more sophisticated dosimetric research, offering valuable new perspectives on the dosimetric benefits of FLASH-RT. A successful transition depends on interdisciplinary research involving biologists, physicists, clinicians, and engineers from lab experiments to patient care. Working as a team, these specialists need to address the technical obstacles in providing FLASH-RT, guaranteeing its accuracy and safety, especially in the intricate anatomical areas. By conducting more dosimetric studies and using improved capabilities of treatment planning systems, the potential of FLASH-RT’s promise to revolutionize tumor treatment is becoming increasingly evident. This progress not only ensures better outcomes for tumor patients but also paves the way for the broader application of FLASH-RT in treating a variety of tumors, thus enhancing overall tumor care. We can anticipate a future when technological expenses will decrease, resulting in FLASH-RT becoming a more attainable choice for clinical application in both human and veterinary radiation oncology. Additionally, we think that with the increasing availability of FLASH-RT data, it could be more accessible. To sum up, while the path for FLASH-RT is challenging, the outlook is optimistic.

## Figures and Tables

**Table 1:** Comparison of FLASH-RT vs. CONV-RT.

Item	CONV-RT	FLASH-RT
Invention time and clinical applicability	1895 (Ubiquitous)	2014 (Nascent)
Equipment	X-knife technique using MV X-rays from a LINAC, Co-60 γ-knife, as well as proton and heavy ion accelerators	LINAC, cyclotron, synchrotron, synchrocyclotron, and laser accelerator
IR source	Proton, electron, X-ray, γ-ray, and heavy ion	Proton, electron, X-ray, and heavy ion
Cost	Less costly strategy	More costly than CONV-RT machines, equipment for X-ray FLASH-RT is cheaper than that required for proton FLASH-RT
Possible RT effect dependencies	Type, size, and location of tumor, duration of treatment, total dose, dose fractionation, radiosensitivity, and oxygen content	Total dose, average dose rate, pulse dose rate, beam-on time, dose rate variations within the target volumes, fractionation, LET, IR source, tissue type, irradiated tissue volume, tissue oxygenation, DNA repair, intrinsic radiosensitivity, and inherent differences in redox and free radical chemistry between normal and tumor tissues
Average dose rate	1–4 Gy/min	≥40 Gy/s
Time delivery	>1 min	<0.1 s
IR-induced toxicity	It has been shown to induce damage to neighboring normal tissues	Many studies have demonstrated that it reduces IR-induced toxicity in normal tissues
Mechanism on normal tissues/tumors	4Rs: DNA damage repair, reoxygenation, repopulation, and cell cycle redistribution	
Tumor control	They have similar antitumor efficacy	

## Appendix A

**Table A1:** Evidence of normal tissue sparing from FLASH-RT.

Models	Site of IR	Assay End Point	Dose (Gy)	Dose Rate (Gy/s)	Beam	Ref.
Mice	WBI	Cognitive testing and novel object recognition testing	10	>100	Electron	[13]
Mice	WBI	Cognitive testing, fear extinction testing, social interaction, social avoidance behaviors, anxiety-like behavior evaluation with the light-dark box test, novel object recognition, and plasma growth hormone quantification	8	4.4 × 10^6^	Electron	[129]
Mice	WBI	Cognitive deficits in memory, dendritic spine density by tracing Golgi-stained hippocampal neurons, evaluation of neuroinflammation by CD68 immunostaining (a marker of activated microglia), and expression of pro-inflammatory cytokines using a multiplex immunoassay	30.4 ± 0.37	200, 300	Electron	[128]
Mice	WBI	Cognitive deficits in memory and learning, and ROS production	3 × 10	>1.8 × 10^6^	Electron	[71]
Mice	WBI	Neurocognitive testing, discrimination index, neuronal morphology, and neuroinflammation	10	>100	Electron	[24]
Mice	WBI	Survival, dermatitis, breathing function, lung pathology, T helper cells pathway modulation, and calcium-induced T lymphocyte apoptosis	15, 17.5, 20	40	Proton	[226]
Mice	Thorax	Survival, lung fibrosis, and skin dermatitis	15, 17.5, 20	40	Proton	[115]
Mice	Thorax	Cellular proliferation, pro-inflammatory genes expression, DNA damage (53BP1/γH2AX foci), senescence, and sparing of lung progenitor cells from IR-induced toxicity	17	40, 60	Electron	[92]
Mice	Thorax	Survival, histopathology, and skin toxicity	10, 16, 20, 30, 40	>180	Electron	[64]
Mice	TAI	Intestinal crypt survival and gut microbiota alterations	7.5, 12.5	≥280	Electron	[139]
Mice	TAI	Survival, gastrointestinal function, epithelial integrity, number of punctate γ-H2AX foci, apoptotic DNA fragmentation, and complete blood count	12, 16	216	Electron	[33]
Mice	TAI	Survival	10, 22	70, 210	Electron	[132]
Mice	TAI	Histopathologic examination of intestinal tissues and survival	13, 22	>40	Proton	[101]
Mice	Skin	Skin toxicity, TGF-β1 production, and changes in cytokine levels in the blood	35	57, 115	Proton	[127]
Rats	Skin	Skin toxicity	8, 12.5, 15	70, 90	Electron	[68]
Mini-pigs	Skin	Skin toxicity (quantification of hair follicle, cutaneous tissue, skin fibro necrosis, and preservation of CD34+ cells defined as epidermal stem cell in the bulge of the hair follicle)	22, 34	≈300	Electron	[15]
Cats	Skin	Mucosal/skin toxicity and overall survival	25, 41	≈300	Electron	[15]
Cats	Nasal planum	Mucosal necrosis and spontaneous fractures	30	1500	Electron	[21]
Zebrafish	Embryo	Survival and morphological alterations, such as pericardial edema and curved spines	20, 23	100	Proton	[30]
Zebrafish	Embryo	Morphometric assessment of neurons and ROS production measurement	8	>100	Electron	[24]
Zebrafish	Embryo	Survival and morphological alterations, such as pericardial edema, spinal curvature, and diameters of the yolk sac and eye	26	10^5^	Electron	[227]
Zebrafish	Embryo	Body-length measurement and morphological alterations, such as pericardial edema and spinal curvature	30	300	Proton	[28]
Human (75-year-old patient with a multiply relapsed cutaneous T-cell lymphoma)	Posterior part of the left arm	Late effects assessment through photographs and skin biopsies	15	166	Electron	[147]
Human (10 patients with bone metastases)	Bone metastases in the extremities	Skin changes, fractures, pain flares, fibrosis, and visible vascular changes in the treatment field	8	≥40	Proton	[36]
Human	Human peripheral blood lymphocyte	Levels of DNA damage and oxygen tension	≥20, ≥30	2 × 10^3^	Electron	[183]

**Table A2:** Evidence of anti-tumor efficacy of FLASH-RT.

Models	Site of IR	Assay End Point	Dose (Gy)	Dose Rate (Gy/s)	IR Modality	Ref.
Mice	WBI	Tumor control	10 14 4 × 3.5 2 × 7 3 × 10	(5.6 × 10^6^, FLASH-RT) (7.8 × 10^6^, FLASH-RT) (1.9 × 10^6^, FLASH-RT) (3.9 × 10^6^, FLASH-RT) (5.6 × 10^6^, FLASH-RT)	Electron	[71]
			10, 14, 4 × 3.5, 2 × 7, 3 × 10	(0.1, CONV-RT)		
Mice	Breast	Survival and tumor size	(18, FLASH-RT) (15, CONV-RT)	(1000, FLASH-RT) (0.1, CONV-RT)	X-ray	[67]
Mice	TAI	Survival and tumor size	30	(700, FLASH-RT) (0.1, CONV-RT)	X-ray	[67]
Mice	TAI	Survival, proliferation, macroscopic tumor burden, and tumor weight	14, 16	(216, FLASH-RT) (0.079, CONV-RT)	Electron	[33]
Mice	TAI	Tumor control	14	(210, FLASH-RT) (0.126, CONV-RT)	Electron	[228]
Mice	TAI	Tumor control	15, 18	(78, FLASH-RT) (0.9, CONV-RT)	Proton	[39]
Mice	TAI	Tumor control	15, 18	(>107, FLASH-RT) (0.82, CONV-RT)	Proton	[121]
Mice	TAI	Tumor nodules quantification and tumor weight	12, 16	(>216, FLASH-RT) (0.079, CONV-RT)	Electron	[33]
Mice	Thorax	Survival	12	(700, FLASH-RT) (0.1, CONV-RT)	X-ray	[67]
Mice	Thorax	Tumors size, recruitment of CD3+, CD4+, and CD8+ cells into the tumor core	18	(40, FLASH-RT) (0.5, CONV-RT)	Proton	[150]
Mice	Thorax	Tumor growth and survival	(16, 30, FLASH-RT) (7.5, 17, CONV-RT)	(≥40, FLASH-RT) (≤0.03, CONV-RT)	Electron	[12]
Mice	Thorax	Vessel morphology, phosphomyosin light chain activation, γ-H2AX foci assessment, intracellular ROS levels, and immune cells infiltration to tumors	15	(352, FLASH-RT) (0.06, CONV-RT)	Electron	[149]
Mice	Limb	Tumor size	40, 60	(83, FLASH-RT) (0.38, CONV-RT)	Proton	[229]
Mice	Limb	Tumor size and metastasis	18	(100, FLASH-RT) (0.3, CONV-RT)	^12^C	[230]
Mice	Limb	Tumor control	30	(69, 124, FLASH-RT) (0.39, 0.65, CONV-RT)	Proton	[76]
Mice	TBI	Tumor growth	4	(200, FLASH-RT) (<0.072, CONV-RT)	Electron	[131]
Mice	Leg	Tumor control	15	(57, 115, FLASH-RT) (1, CONV-RT)	Proton	[127]
Rats	Skin	Survival and tumor growth	8, 12.5, 15	(70, 90, FLASH-RT) (~0.1, CONV-RT)	Electron	[68]
Cats	Skin	Tumor control	25, 41	(≈300, FLASH-RT) (≈0.08, CONV-RT)	Electron	[15]

**Table A3:** In vitro studies of the differential response of FLASH-RT.

Cell Culture and Tumor Model	Assay End Point	Dose (Gy)	Dose Rate (Gy/s)	IR Source	Ref.
Chinese hamster V79 lung embryonic fibroblasts, human squamous carcinoma SQ-20B cells, and human colon adenocarcinoma LoVo cells	Clonogenic survival, apoptosis, and mitotic cell death	7.5	120	Electron	[26]
V-79 Chinese hamster live cell line	Clonogenic survival	5	>10^9^	Proton	[148]
Chinese hamster ovary cells (CHO-K1)	Oxygen consumption	7.5	70	^12^C	[231]
Murine GBM H454 cell line	Clonogenic survival, neuronal morphology, dendritic spine density, and neuroinflammation	20	>100	Electron	[24]
Human lung fibroblast cell lines (MRC5 and IMR-90)	Immunofluorescent determination of IR-induced foci of the 53BP1/γH2AX proteins at sites of DNA damage, monitoring cell proliferation, and senescence in mouse lung	4, 5	40, 60	Electron	[92]
Normal human lung fibroblasts (HFL1), and human salivary gland cancer cells (HSGc-C5)	Survival, proliferation, and senescence	1, 2, 3	96, 195	^12^C	[69]
Lung adenocarcinoma spheroids (A549)	Clonogenic survival, cell viability, and spheroid growth	5, 10, 15, 20	90	Electron	[38]
Human-hamster hybrid (AL) cell line	Chromosome aberrations	3.6	–	Proton	[63]
Human HeLa cells (subtype HeLa-RIKEN)	G2 phase cell cycle arrest, apoptosis, and colony formation	3	>10^9^	Proton	[232]
Human peripheral blood lymphocyte	Levels of DNA damage and oxygen tension	20	2 × 10^3^	Electron	[183]
Normal diploid human lung fibroblasts (IMR90)	Clonogenic cell survival, γH2AX foci formation, induction of premature senescence, and the expression of the pro-inflammatory marker TGF-β	10, 20	100, 1000	Proton	[114]
Human breast cancer cells (MDA-MB-231, MCF7), human colon cancer cell line (WiDr), squamous cell carcinoma cell line (LU-HNSCC4), HeLa cell line, and normal lung fibroblasts (MRC-5)	Survival and IR-induced DNA-damage foci using a 53BP1-marker	6, 10	≥800	Electron	[233]
Prostate cancer cells	Survival, oxygen consumption, and radiolytic ROS production	150	10^9^	Electron	[234]
Human prostate cancer cells (DU145)	Survival under normoxic and hypoxic conditions	18	600	Electron	[50]
Mammalian cells	Oxygen consumption	30	100	Electron	[91]

**Table A4:** In vivo studies of the differential response of FLASH-RT.

Models	Site of IR	Assay End Point	Dose (Gy)	Dose Rate (Gy/s)	IR Source	Ref.
Mice	WBI	Oxygen consumption	30	100	Electron	[91]
Mice	WBI	Analysis of astrogliosis and immune signaling in the brain	10	5.6 × 10^6^	Electron	[235]
Mice	TAI	Survival	25	83	Electron	[7]
Mice	TAI	Survival	17.5	70, 210	Electron	[132]
Mice	TAI	Intestinal crypt cell proliferation and intestinal fibrosis	16.6, 18.3	78	Proton	[39]
Mice	TAI	Survival, epithelial integrity, intestinal function, DNA repair in intestinal CBC cells using γ-H2AX immunofluorescence, proliferation, and apoptosis	14, 25	>1.8 × 10^6^	Electron	[33]
Mice	TAI	Intestinal regeneration, T-regulatory cells, and cytolytic CD8+ T cells in tumor microenvironment (TME)	14	210	Electron	[228]
Mice	Thorax	TNF-α activation, lung fibrogenesis, apoptosis, and sparing of epithelial cells and normal smooth muscle	16, 30	≥40	Electron	[12]
Mice	–	Oxygen-depletion quantification	30	300	Electron	[164]
Mice	Limb	Skin damage and IR-induced fibrosis	40, 60	83	Proton	[229]
Mice	Limb	Structural changes in limb muscle	18	100	^12^C	[230]
Mice	Limb	Inflammation, TGFβ1 expression, and skin reaction	30	69, 124	Proton	[76]
Mini-pig and cats	Skin	Skin toxicity	22, 34	≈300	Electron	[15]

## References

[1] Baumann M., Krause M., Overgaard J., Debus J., Bentzen S.M., Daartz J., Richter C., Zips D., Bortfeld T. (2016). Radiation oncology in the era of precision medicine. Nat. Rev Cancer.

[2] Bourhis J., Sozzi W.J., Jorge P.G., Gaide O., Bailat C., Duclos F., Patin D., Ozsahin M., Bochud F., Germond J.F. (2019). Treatment of a first patient with FLASH-radiotherapy. Radiother. Oncol..

[3] Lin B., Gao F., Yang Y., Wu D., Zhang Y., Feng G., Dai T., Du X. (2021). FLASH Radiotherapy: History and Future. Front. Oncol..

[4] Smyth L.M.L., Donoghue J.F., Ventura J.A., Livingstone J., Bailey T., Day L.R.J., Crosbie J.C., Rogers P.A.W. (2018). Comparative toxicity of synchrotron and conventional radiation therapy based on total and partial body irradiation in a murine model. Sci. Rep..

[5] Hughes J.R., Parsons J.L. (2020). FLASH Radiotherapy: Current Knowledge and Future Insights Using Proton-Beam Therapy. Int. J. Mol. Sci..

[6] Liew H., Mein S., Dokic I., Haberer T., Debus J., Abdollahi A., Mairani A. (2021). Deciphering Time-Dependent DNA Damage Complexity, Repair, and Oxygen Tension: A Mechanistic Model for FLASH-Dose-Rate Radiation Therapy. Int. J. Radiat. Oncol. Biol. Phys..

[7] Hornsey S., Bewley D.K. (1971). Hypoxia in mouse intestine induced by electron irradiation at high dose-rates. Int. J. Radiat. Biol. Relat. Stud. Phys. Chem. Med..

[8] Dewey D.L., Boag J.W. (1959). Modification of the oxygen effect when bacteria are given large pulses of radiation. Nature.

[9] Epp E.R., Weiss H., Djordjevic B., Santomasso A. (1972). The Radiosensitivity of Cultured Mammalian Cells Exposed to Single High Intensity Pulses of Electrons in Various Concentrations of Oxygen. Radiat. Res..

[10] Weiss H., Epp E.R., Heslin J.M., Ling C.C., Santomasso A. (1974). Oxygen depletion in cells irradiated at ultra-high dose-rates and at conventional dose-rates. Int. J. Radiat. Biol. Relat. Stud. Phys. Chem. Med..

[11] Bourhis J., Montay-Gruel P., Goncalves Jorge P., Bailat C., Petit B., Ollivier J., Jeanneret-Sozzi W., Ozsahin M., Bochud F., Moeckli R. (2019). Clinical translation of FLASH radiotherapy: Why and how?. Radiother. Oncol..

[12] Favaudon V., Caplier L., Monceau V., Pouzoulet F., Sayarath M., Fouillade C., Poupon M.F., Brito I., Hupe P., Bourhis J. (2014). Ultrahigh dose-rate FLASH irradiation increases the differential response between normal and tumor tissue in mice. Sci. Transl. Med..

[13] Montay-Gruel P., Petersson K., Jaccard M., Boivin G., Germond J.-F., Petit B., Doenlen R., Favaudon V., Bochud F., Bailat C. (2017). Irradiation in a flash: Unique sparing of memory in mice after whole brain irradiation with dose rates above 100 Gy/s. Radiother. Oncol..

[14] Yoganathan S.A., Maria Das K.J., Agarwal A., Kumar S. (2017). Magnitude, impact, and management of respiration-induced target motion in radiotherapy treatment: A comprehensive review. J. Med. Phys..

[15] Vozenin M.C., De Fornel P., Petersson K., Favaudon V., Jaccard M., Germond J.F., Petit B., Burki M., Ferrand G., Patin D. (2019). The Advantage of FLASH Radiotherapy Confirmed in Mini-pig and Cat-cancer Patients. Clin. Cancer Res..

[16] Kickhefel A., Rosenberg C., Roland J., Viallon M., Gross P., Schick F., Hosten N., Salomir R. (2012). A pilot study for clinical feasibility of the near-harmonic 2D referenceless PRFS thermometry in liver under free breathing using MR-guided LITT ablation data. Int. J. Hyperth..

[17] Montay-Gruel P., Bouchet A., Jaccard M., Patin D., Serduc R., Aim W., Petersson K., Petit B., Bailat C., Bourhis J. (2018). X-rays can trigger the FLASH effect: Ultra-high dose-rate synchrotron light source prevents normal brain injury after whole brain irradiation in mice. Radiother. Oncol..

[18] Jaccard M., Duran M.T., Petersson K., Germond J.F., Liger P., Vozenin M.C., Bourhis J., Bochud F., Bailat C. (2018). High dose-per-pulse electron beam dosimetry: Commissioning of the Oriatron eRT6 prototype linear accelerator for preclinical use. Med. Phys..

[19] Schuler E., Trovati S., King G., Lartey F., Rafat M., Villegas M., Praxel A.J., Loo B.W., Maxim P.G. (2017). Experimental Platform for Ultra-high Dose Rate FLASH Irradiation of Small Animals Using a Clinical Linear Accelerator. Int. J. Radiat. Oncol. Biol. Phys..

[20] Rahman M., Ashraf M.R., Zhang R., Bruza P., Dexter C.A., Thompson L., Cao X., Williams B.B., Hoopes P.J., Pogue B.W. (2021). Electron FLASH Delivery at Treatment Room Isocenter for Efficient Reversible Conversion of a Clinical LINAC. Int. J. Radiat. Oncol. Biol. Phys..

[21] Rohrer Bley C., Wolf F., Goncalves Jorge P., Grilj V., Petridis I., Petit B., Bohlen T.T., Moeckli R., Limoli C., Bourhis J. (2022). Dose- and Volume-Limiting Late Toxicity of FLASH Radiotherapy in Cats with Squamous Cell Carcinoma of the Nasal Planum and in Mini Pigs. Clin. Cancer Res..

[22] Wilson J.D., Hammond E.M., Higgins G.S., Petersson K. (2019). Ultra-High Dose Rate (FLASH) Radiotherapy: Silver Bullet or Fool’s Gold?. Front. Oncol..

[23] Maxim P.G., Tantawi S.G., Loo B.W. (2019). PHASER: A platform for clinical translation of FLASH cancer radiotherapy. Radiother. Oncol..

[24] Montay-Gruel P., Acharya M.M., Petersson K., Alikhani L., Yakkala C., Allen B.D., Ollivier J., Petit B., Jorge P.G., Syage A.R. (2019). Long-term neurocognitive benefits of FLASH radiotherapy driven by reduced reactive oxygen species. Proc. Natl. Acad. Sci. USA.

[25] Fernet M., Ponette V., Deniaud-Alexandre E., Menissier-De Murcia J., De Murcia G., Giocanti N., Megnin-Chanet F., Favaudon V. (2000). Poly(ADP-ribose) polymerase, a major determinant of early cell response to ionizing radiation. Int. J. Radiat. Biol..

[26] Ponette V., Le Pechoux C., Deniaud-Alexandre E., Fernet M., Giocanti N., Tourbez H., Favaudon V. (2000). Hyperfast, early cell response to ionizing radiation. Int. J. Radiat. Biol..

[27] Friedl A.A., Prise K.M., Butterworth K.T., Montay-Gruel P., Favaudon V. (2022). Radiobiology of the FLASH effect. Med. Phys..

[28] Karsch L., Pawelke J., Brand M., Hans S., Hideghety K., Jansen J., Lessmann E., Lock S., Schurer M., Schurig R. (2022). Beam pulse structure and dose rate as determinants for the flash effect observed in zebrafish embryo. Radiother. Oncol..

[29] Zou W., Zhang R., Schuler E., Taylor P.A., Mascia A.E., Diffenderfer E.S., Zhao T., Ayan A.S., Sharma M., Yu S.J. (2023). Framework for Quality Assurance of Ultrahigh Dose Rate Clinical Trials Investigating FLASH Effects and Current Technology Gaps. Int. J. Radiat. Oncol. Biol. Phys..

[30] Beyreuther E., Brand M., Hans S., Hideghety K., Karsch L., Lessmann E., Schurer M., Szabo E.R., Pawelke J. (2019). Feasibility of proton FLASH effect tested by zebrafish embryo irradiation. Radiother. Oncol..

[31] Beyreuther E., Karsch L., Laschinsky L., Lessmann E., Naumburger D., Oppelt M., Richter C., Schurer M., Woithe J., Pawelke J. (2015). Radiobiological response to ultra-short pulsed megavoltage electron beams of ultra-high pulse dose rate. Int. J. Radiat. Biol..

[32] Vozenin M.C., Hendry J.H., Limoli C.L. (2019). Biological Benefits of Ultra-high Dose Rate FLASH Radiotherapy: Sleeping Beauty Awoken. Clin. Oncol. (R. Coll. Radiol.).

[33] Levy K., Natarajan S., Wang J., Chow S., Eggold J.T., Loo P.E., Manjappa R., Melemenidis S., Lartey F.M., Schuler E. (2020). Abdominal FLASH irradiation reduces radiation-induced gastrointestinal toxicity for the treatment of ovarian cancer in mice. Sci. Rep..

[34] Schulte R., Johnstone C., Boucher S., Esarey E., Geddes C.G.R., Kravchenko M., Kutsaev S., Loo B.W., Meot F., Mustapha B. (2023). Transformative Technology for FLASH Radiation Therapy. Appl. Sci..

[35] Matuszak N., Suchorska W.M., Milecki P., Kruszyna-Mochalska M., Misiarz A., Pracz J., Malicki J. (2022). FLASH radiotherapy: An emerging approach in radiation therapy. Rep. Pract. Oncol. Radiother..

[36] Mascia A.E., Daugherty E.C., Zhang Y., Lee E., Xiao Z., Sertorio M., Woo J., Backus L.R., McDonald J.M., McCann C. (2023). Proton FLASH Radiotherapy for the Treatment of Symptomatic Bone Metastases: The FAST-01 Nonrandomized Trial. JAMA Oncol..

[37] Hendry J.H., Moore J.V., Hodgson B.W., Keene J.P. (1982). The Constant Low Oxygen Concentration in All the Target Cells for Mouse Tail Radionecrosis. Radiat. Res..

[38] Khan S., Bassenne M., Wang J., Manjappa R., Melemenidis S., Breitkreutz D.Y., Maxim P.G., Xing L., Loo B.W., Pratx G. (2021). Multicellular Spheroids as In Vitro Models of Oxygen Depletion During FLASH Irradiation. Int. J. Radiat. Oncol. Biol. Phys..

[39] Diffenderfer E.S., Verginadis I.I., Kim M.M., Shoniyozov K., Velalopoulou A., Goia D., Putt M., Hagan S., Avery S., Teo K. (2020). Design, Implementation, and in Vivo Validation of a Novel Proton FLASH Radiation Therapy System. Int. J. Radiat. Oncol. Biol. Phys..

[40] Somorin O., Ameghashitsi L. (1987). Trypsin catalyzed hydrolysis of new chromogenic arginine substrates. Biochem. Int..

[41] Vozenin M.C., Bourhis J., Durante M. (2022). Towards clinical translation of FLASH radiotherapy. Nat. Rev. Clin. Oncol..

[42] Verhaegen F., Wanders R.G., Wolfs C., Eekers D. (2021). Considerations for shoot-through FLASH proton therapy. Phys. Med. Biol..

[43] Lempart M., Blad B., Adrian G., Back S., Knoos T., Ceberg C., Petersson K. (2019). Modifying a clinical linear accelerator for delivery of ultra-high dose rate irradiation. Radiother. Oncol..

[44] Weber U.A., Scifoni E., Durante M. (2022). FLASH radiotherapy with carbon ion beams. Med. Phys..

[45] Patriarca A., Fouillade C., Auger M., Martin F., Pouzoulet F., Nauraye C., Heinrich S., Favaudon V., Meyroneinc S., Dendale R. (2018). Experimental Set-up for FLASH Proton Irradiation of Small Animals Using a Clinical System. Int. J. Radiat. Oncol. Biol. Phys..

[46] Vozenin M.C., Montay-Gruel P., Limoli C., Germond J.F. (2020). All Irradiations that are Ultra-High Dose Rate may not be FLASH: The Critical Importance of Beam Parameter Characterization and In Vivo Validation of the FLASH Effect. Radiat. Res..

[47] Pratx G., Kapp D.S. (2019). A computational model of radiolytic oxygen depletion during FLASH irradiation and its effect on the oxygen enhancement ratio. Phys. Med. Biol..

[48] Pratx G., Kapp D.S. (2019). Ultra-High-Dose-Rate FLASH Irradiation May Spare Hypoxic Stem Cell Niches in Normal Tissues. Int. J. Radiat. Oncol. Biol. Phys..

[49] Zhang Q., Gerweck L.E., Cascio E., Yang Q., Huang P., Niemierko A., Bertolet A., Nesteruk K.P., McNamara A., Schuemann J. (2023). Proton FLASH effects on mouse skin at different oxygen tensions. Phys. Med. Biol..

[50] Adrian G., Konradsson E., Lempart M., Back S., Ceberg C., Petersson K. (2020). The FLASH effect depends on oxygen concentration. Br. J. Radiol..

[51] Taylor E., Hill R.P., Letourneau D. (2022). Modeling the impact of spatial oxygen heterogeneity on radiolytic oxygen depletion during FLASH radiotherapy. Phys Med Biol.

[52] Berry R.J. (1973). Effects of radiation dose-rate from protracted, continuous irradiation to ultra-high dose-rates from pulsed accelerators. Br. Med. Bull..

[53] Hornsey S., Alper T. (1966). Unexpected dose-rate effect in the killing of mice by radiation. Nature.

[54] Town C.D. (1967). Radiobiology. Effect of high dose rates on survival of mammalian cells. Nature.

[55] Todd P., Winchell H.S., Feola J.M., Jones G.E. (1968). Pulsed high-intensity roentgen rays. Inactivation of human cells cultured in vitro and limitations on usefulness in radiotherapy. Acta Radiol. Ther. Phys. Biol..

[56] Oliver R. (1969). The effect of irradiation at extremely high dose-rates. Br. J. Radiol..

[57] Berry R.J., Hall E.J., Forster D.W., Storr T.H., Goodman M.J. (1969). Survival of mammalian cells exposed to x rays at ultra-high dose-rates. Br. J. Radiol..

[58] Field S.B., Bewley D.K. (1974). Effects of dose-rate on the radiation response of rat skin. Int. J. Radiat. Biol. Relat. Stud. Phys. Chem. Med..

[59] Nias A.H., Swallow A.J., Keene J.P., Hodgson B.W. (1970). Survival of HeLa cells from 10 nanosecond pulses of electrons. Int. J. Radiat. Biol. Relat. Stud. Phys. Chem. Med..

[60] Prempree T., Michelsen A., Merz T. (1969). The repair time of chromosome breaks induced by pulsed x-rays on ultra-high dose-rate. Int. J. Radiat. Biol. Relat. Stud. Phys. Chem. Med..

[61] Purrott R.J., Reeder E.J. (1977). Chromosome aberration yields induced in human lymphocytes by 15 MeV electrons given at a conventional dose-rate and in microsecond pulses. Int. J. Radiat. Biol. Relat. Stud. Phys. Chem. Med..

[62] Martin M. (2009). Laser accelerated radiotherapy: Is it on its way to the clinic?. J. Natl. Cancer Inst..

[63] Schmid T.E., Dollinger G., Hable V., Greubel C., Zlobinskaya O., Michalski D., Auer S., Friedl A.A., Schmid E., Molls M. (2011). The Effectiveness of 20 MeV Protons at Nanosecond Pulse Lengths in Producing Chromosome Aberrations in Human-Hamster Hybrid Cells. Radiat. Res..

[64] Soto L.A., Casey K.M., Wang J., Blaney A., Manjappa R., Breitkreutz D., Skinner L., Dutt S., Ko R.B., Bush K. (2020). FLASH Irradiation Results in Reduced Severe Skin Toxicity Compared to Conventional-Dose-Rate Irradiation. Radiat. Res..

[65] Jin J.Y., Gu A., Wang W., Oleinick N.L., Machtay M., Spring Kong F.M. (2020). Ultra-high dose rate effect on circulating immune cells: A potential mechanism for FLASH effect?. Radiother. Oncol..

[66] Konradsson E., Arendt M.L., Bastholm Jensen K., Borresen B., Hansen A.E., Back S., Kristensen A.T., Munck Af Rosenschold P., Ceberg C., Petersson K. (2021). Establishment and Initial Experience of Clinical FLASH Radiotherapy in Canine Cancer Patients. Front. Oncol..

[67] Gao F., Yang Y., Zhu H., Wang J., Xiao D., Zhou Z., Dai T., Zhang Y., Feng G., Li J. (2022). First demonstration of the FLASH effect with ultrahigh dose rate high-energy X-rays. Radiother. Oncol..

[68] Konradsson E., Liljedahl E., Gustafsson E., Adrian G., Beyer S., Ilaahi S.E., Petersson K., Ceberg C., Nittby Redebrandt H. (2022). Comparable Long-Term Tumor Control for Hypofractionated FLASH Versus Conventional Radiation Therapy in an Immunocompetent Rat Glioma Model. Adv. Radiat. Oncol..

[69] Tashiro M., Yoshida Y., Oike T., Nakao M., Yusa K., Hirota Y., Ohno T. (2022). First Human Cell Experiments with FLASH Carbon Ions. Anticancer Res..

[70] Daugherty E.C., Mascia A., Zhang Y., Lee E., Xiao Z., Sertorio M., Woo J., McCann C., Russell K., Levine L. (2023). FLASH Radiotherapy for the Treatment of Symptomatic Bone Metastases (FAST-01): Protocol for the First Prospective Feasibility Study. JMIR Res. Protoc..

[71] Montay-Gruel P., Acharya M.M., Goncalves Jorge P., Petit B., Petridis I.G., Fuchs P., Leavitt R., Petersson K., Gondre M., Ollivier J. (2021). Hypofractionated FLASH-RT as an Effective Treatment against Glioblastoma that Reduces Neurocognitive Side Effects in Mice. Clin. Cancer Res..

[72] Huang C.C., Mendonca M.S. (2021). News FLASH-RT: To Treat GBM and Spare Cognition, Fraction Size and Total Dose Matter. Clin. Cancer Res..

[73] Källén K. (2021). Toxicity of Pulsed Beams in Radiation Therapy from a Physio-Chemical Perspective. Bachelor’s Thesis.

[74] Montay-Gruel P., Corde S., Laissue J.A., Bazalova-Carter M. (2022). FLASH radiotherapy with photon beams. Med. Phys..

[75] Folkerts M.M., Abel E., Busold S., Perez J.R., Krishnamurthi V., Ling C.C. (2020). A framework for defining FLASH dose rate for pencil beam scanning. Med. Phys..

[76] Velalopoulou A., Karagounis I.V., Cramer G.M., Kim M.M., Skoufos G., Goia D., Hagan S., Verginadis I.I., Shoniyozov K., Chiango J. (2021). FLASH Proton Radiotherapy Spares Normal Epithelial and Mesenchymal Tissues While Preserving Sarcoma Response. Cancer Res.

[77] Aridgides P., Bogart J. (2016). Stereotactic Body Radiation Therapy for Stage I Non–Small Cell Lung Cancer. Thorac. Surg. Clin..

[78] Macià i Garau M. (2017). Radiobiology of stereotactic body radiation therapy (SBRT). Rep. Pract. Oncol. Radiother..

[79] Wang K., Mavroidis P., Royce T.J., Falchook A.D., Collins S.P., Sapareto S., Sheets N.C., Fuller D.B., El Naqa I., Yorke E. (2021). Prostate Stereotactic Body Radiation Therapy: An Overview of Toxicity and Dose Response. Int. J. Radiat. Oncol. Biol. Phys..

[80] Benedict S.H., Yenice K.M., Followill D., Galvin J.M., Hinson W., Kavanagh B., Keall P., Lovelock M., Meeks S., Papiez L. (2010). Stereotactic body radiation therapy: The report of AAPM Task Group 101. Med. Phys..

[81] van Marlen P., Verbakel W.F.A.R., Slotman B.J., Dahele M. (2022). Single-fraction 34 Gy Lung Stereotactic Body Radiation Therapy Using Proton Transmission Beams: FLASH-dose Calculations and the Influence of Different Dose-rate Methods and Dose/Dose-rate Thresholds. Adv. Radiat. Oncol..

[82] Kinj R., Muggeo E., Schiappacasse L., Bourhis J., Herrera F.G. (2022). Stereotactic Body Radiation Therapy in Patients with Oligometastatic Disease: Clinical State of the Art and Perspectives. Cancers.

[83] Choi H.S., Jeong B.K., Kang K.M., Jeong H., Song J.H., Ha I.B., Kwon O.-Y. (2020). Tumor Control and Overall Survival after Stereotactic Body Radiotherapy for Pulmonary Oligometastases from Colorectal Cancer: A Meta-Analysis. Cancer Res. Treat..

[84] Videtic G.M.M., Stephans K.L., Woody N.M., Reddy C.A., Zhuang T., Magnelli A., Djemil T. (2014). 30 Gy or 34 Gy? Comparing 2 Single-Fraction SBRT Dose Schedules for Stage I Medically Inoperable Non-Small Cell Lung Cancer. Int. J. Radiat. Oncol. Biol. Phys..

[85] Chang B.K., Timmerman R.D. (2007). Stereotactic Body Radiation Therapy. Am. J. Clin. Oncol..

[86] Katano A., Minamitani M., Ohira S., Yamashita H. (2024). Recent Advances and Challenges in Stereotactic Body Radiotherapy. Technol. Cancer Res. Treat..

[87] Lo S.S., Sahgal A., Chang E.L., Mayr N.A., Teh B.S., Huang Z., Schefter T.E., Yao M., Machtay M., Slotman B.J. (2013). Serious Complications Associated with Stereotactic Ablative Radiotherapy and Strategies to Mitigate the Risk. Clin. Oncol..

[88] Hodge W., Tomé W.A., Jaradat H.A., Orton N.P., Khuntia D., Traynor A., Weigel T., Mehta M.P. (2009). Feasibility report of image guided stereotactic body radiotherapy (IG-SBRT) with tomotherapy for early stage medically inoperable lung cancer using extreme hypofractionation. Acta Oncol..

[89] van der Voort van Zyp N.C., Prévost J.-B., Hoogeman M.S., Praag J., van der Holt B., Levendag P.C., van Klaveren R.J., Pattynama P., Nuyttens J.J. (2009). Stereotactic radiotherapy with real-time tumor tracking for non-small cell lung cancer: Clinical outcome. Radiother. Oncol..

[90] Hoogeman M.S., Nuyttens J.J., Levendag P.C., Heijmen B.J.M. (2008). Time Dependence of Intrafraction Patient Motion Assessed by Repeat Stereoscopic Imaging. Int. J. Radiat. Oncol. Biol. Phys..

[91] Petersson K., Adrian G., Butterworth K., McMahon S.J. (2020). A Quantitative Analysis of the Role of Oxygen Tension in FLASH Radiation Therapy. Int. J. Radiat. Oncol. Biol. Phys..

[92] Fouillade C., Curras-Alonso S., Giuranno L., Quelennec E., Heinrich S., Bonnet-Boissinot S., Beddok A., Leboucher S., Karakurt H.U., Bohec M. (2020). FLASH Irradiation Spares Lung Progenitor Cells and Limits the Incidence of Radio-induced Senescence. Clin. Cancer Res..

[93] Tada E., Parent J.M., Lowenstein D.H., Fike J.R. (2000). X-irradiation causes a prolonged reduction in cell proliferation in the dentate gyrus of adult rats. Neuroscience.

[94] Balentova S., Adamkov M. (2015). Molecular, Cellular and Functional Effects of Radiation-Induced Brain Injury: A Review. Int. J. Mol. Sci..

[95] Rola R., Raber J., Rizk A., Otsuka S., VandenBerg S.R., Morhardt D.R., Fike J.R. (2004). Radiation-induced impairment of hippocampal neurogenesis is associated with cognitive deficits in young mice. Exp. Neurol..

[96] Mali S.B., Dahivelkar S. (2023). Flash radiotherapy-gateway to promised land or another mirage. Oral Oncol..

[97] Berry R.J., Stedeford J.B. (1972). Reproductive survival of mammalian cells after irradiation at ultra-high dose-rates: Further observations and their importance for radiotherapy. Br. J. Radiol..

[98] Ling C.C., Michaels H.B., Epp E.R., Peterson E.C. (1978). Oxygen Diffusion into Mammalian Cells Following Ultrahigh Dose Rate Irradiation and Lifetime Estimates of Oxygen-Sensitive Species. Radiation Research.

[99] Liew H., Mein S., Tessonnier T., Abdollahi A., Debus J., Dokic I., Mairani A. (2023). Do We Preserve Tumor Control Probability (TCP) in FLASH Radiotherapy? A Model-Based Analysis. Int. J. Mol. Sci..

[100] Goncalves Jorge P., Grilj V., Bourhis J., Vozenin M.C., Germond J.F., Bochud F., Bailat C., Moeckli R. (2022). Technical note: Validation of an ultrahigh dose rate pulsed electron beam monitoring system using a current transformer for FLASH preclinical studies. Med. Phys..

[101] Zhang Q., Cascio E., Li C., Yang Q., Gerweck L.E., Huang P., Gottschalk B., Flanz J., Schuemann J. (2020). FLASH Investigations Using Protons: Design of Delivery System, Preclinical Setup and Confirmation of FLASH Effect with Protons in Animal Systems. Radiat. Res..

[102] Jorge P.G., Jaccard M., Petersson K., Gondre M., Duran M.T., Desorgher L., Germond J.F., Liger P., Vozenin M.C., Bourhis J. (2019). Dosimetric and preparation procedures for irradiating biological models with pulsed electron beam at ultra-high dose-rate. Radiother. Oncol..

[103] Felici G., Barca P., Barone S., Bortoli E., Borgheresi R., De Stefano S., Di Francesco M., Grasso L., Linsalata S., Marfisi D. (2020). Transforming an IORT Linac Into a FLASH Research Machine: Procedure and Dosimetric Characterization. Front. Phys..

[104] Bazalova-Carter M., Qu B., Palma B., Hårdemark B., Hynning E., Jensen C., Maxim P.G., Loo B.W. (2015). Treatment planning for radiotherapy with very high-energy electron beams and comparison of VHEE and VMAT plans. Med. Phys..

[105] Bazalova-Carter M., Esplen N. (2019). On the capabilities of conventional X-ray tubes to deliver ultra-high (FLASH) dose rates. Med. Phys..

[106] Cecchi D.D., Therriault-Proulx F., Lambert-Girard S., Hart A., Macdonald A., Pfleger M., Lenckowski M., Bazalova-Carter M. (2021). Characterization of an X-ray tube-based ultrahigh dose-rate system for in vitro irradiations. Med. Phys..

[107] Cheng C., Xu L., Jing H., Selvaraj B., Lin H., Pennock M., Chhabra A.M., Hasan S., Zhai H., Zhang Y. (2024). The Potential and Challenges of Proton FLASH in Head and Neck Cancer Reirradiation. Cancers.

[108] Jakobi A., Bandurska-Luque A., Stützer K., Haase R., Löck S., Wack L.-J., Mönnich D., Thorwarth D., Perez D., Lühr A. (2015). Identification of Patient Benefit From Proton Therapy for Advanced Head and Neck Cancer Patients Based on Individual and Subgroup Normal Tissue Complication Probability Analysis. Int. J. Radiat. Oncol. Biol. Phys..

[109] Colaco R.J., Hoppe B.S., Flampouri S., McKibben B.T., Henderson R.H., Bryant C., Nichols R.C., Mendenhall W.M., Li Z., Su Z. (2015). Rectal Toxicity After Proton Therapy For Prostate Cancer: An Analysis of Outcomes of Prospective Studies Conducted at the University of Florida Proton Therapy Institute. Int. J. Radiat. Oncol. Biol. Phys..

[110] Leroy R., Benahmed N., Hulstaert F., Van Damme N., De Ruysscher D. (2016). Proton Therapy in Children: A Systematic Review of Clinical Effectiveness in 15 Pediatric Cancers. Int. J. Radiat. Oncol. Biol. Phys..

[111] Paganetti H., Niemierko A., Ancukiewicz M., Gerweck L.E., Goitein M., Loeffler J.S., Suit H.D. (2002). Relative biological effectiveness (RBE) values for proton beam therapy. Int. J. Radiat. Oncol. Biol. Phys..

[112] van Marlen P., Dahele M., Folkerts M., Abel E., Slotman B.J., Verbakel W.F.A.R. (2020). Bringing FLASH to the Clinic: Treatment Planning Considerations for Ultrahigh Dose-Rate Proton Beams. Int. J. Radiat. Oncol. Biol. Phys..

[113] Britten R.A., Nazaryan V., Davis L.K., Klein S.B., Nichiporov D., Mendonca M.S., Wolanski M., Nie X., George J., Keppel C. (2013). Variations in the RBE for cell killing along the depth-dose profile of a modulated proton therapy beam. Radiat. Res..

[114] Buonanno M., Grilj V., Brenner D.J. (2019). Biological effects in normal cells exposed to FLASH dose rate protons. Radiother. Oncol..

[115] Girdhani S., Abel E., Katsis A., Rodriquez A., Senapati S., KuVillanueva A., Jackson I.L., Eley J., Vujaskovic Z., Parry R. (2019). Abstract LB-280: FLASH: A novel paradigm changing tumor irradiation platform that enhances therapeutic ratio by reducing normal tissue toxicity and activating immune pathways. Cancer Res..

[116] Fenwick J.D., Mayhew C., Jolly S., Amos R.A., Hawkins M.A. (2024). Navigating the straits: Realizing the potential of proton FLASH through physics advances and further pre-clinical characterization. Front. Oncol..

[117] Kang M., Wei S., Choi J.I., Simone C.B., Lin H. (2021). Quantitative Assessment of 3D Dose Rate for Proton Pencil Beam Scanning FLASH Radiotherapy and Its Application for Lung Hypofractionation Treatment Planning. Cancers.

[118] Kang M., Wei S., Choi J.I., Lin H., Simone C.B. (2022). A Universal Range Shifter and Range Compensator Can Enable Proton Pencil Beam Scanning Single-Energy Bragg Peak FLASH-RT Treatment Using Current Commercially Available Proton Systems. Int. J. Radiat. Oncol. Biol. Phys..

[119] Schüller A., Heinrich S., Fouillade C., Subiel A., De Marzi L., Romano F., Peier P., Trachsel M., Fleta C., Kranzer R. (2020). The European Joint Research Project UHDpulse—Metrology for advanced radiotherapy using particle beams with ultra-high pulse dose rates. Phys. Medica.

[120] Jolly S., Owen H., Schippers M., Welsch C. (2020). Technical challenges for FLASH proton therapy. Phys. Medica.

[121] Kim M.M., Verginadis I.I., Goia D., Haertter A., Shoniyozov K., Zou W., Maity A., Busch T.M., Metz J.M., Cengel K.A. (2021). Comparison of FLASH Proton Entrance and the Spread-Out Bragg Peak Dose Regions in the Sparing of Mouse Intestinal Crypts and in a Pancreatic Tumor Model. Cancers.

[122] Zhang G., Gao W., Peng H. (2022). Design of static and dynamic ridge filters for FLASH–IMPT: A simulation study. Med. Phys..

[123] Roddy D., Bélanger-Champagne C., Tattenberg S., Yen S., Trinczek M., Hoehr C. (2024). Design, optimization, and testing of ridge filters for proton FLASH radiotherapy at TRIUMF: The HEDGEHOG. Nucl. Instrum. Methods Phys. Res. Sect. A Accel. Spectrometers Detect. Assoc. Equip..

[124] Ringbæk T.P., Weber U., Santiago A., Iancu G., Wittig A., Grzanka L., Bassler N., Engenhart-Cabillic R., Zink K. (2018). Validation of new 2D ripple filters in proton treatments of spherical geometries and non-small cell lung carcinoma cases. Phys. Med. Biol..

[125] Evans T., Cooley J., Wagner M., Yu T., Zwart T. (2022). Demonstration of the FLASH Effect Within the Spread-out Bragg Peak After Abdominal Irradiation of Mice. Int. J. Part. Ther..

[126] Pennock M., Wei S., Cheng C., Lin H., Hasan S., Chhabra A.M., Choi J.I., Bakst R.L., Kabarriti R., Simone C.B. (2023). Proton Bragg Peak FLASH Enables Organ Sparing and Ultra-High Dose-Rate Delivery: Proof of Principle in Recurrent Head and Neck Cancer. Cancers.

[127] Cunningham S., McCauley S., Vairamani K., Speth J., Girdhani S., Abel E., Sharma R.A., Perentesis J.P., Wells S.I., Mascia A. (2021). FLASH Proton Pencil Beam Scanning Irradiation Minimizes Radiation-Induced Leg Contracture and Skin Toxicity in Mice. Cancers.

[128] Simmons D.A., Lartey F.M., Schuler E., Rafat M., King G., Kim A., Ko R., Semaan S., Gonzalez S., Jenkins M. (2019). Reduced cognitive deficits after FLASH irradiation of whole mouse brain are associated with less hippocampal dendritic spine loss and neuroinflammation. Radiother. Oncol..

[129] Alaghband Y., Cheeks S.N., Allen B.D., Montay-Gruel P., Doan N.L., Petit B., Jorge P.G., Giedzinski E., Acharya M.M., Vozenin M.C. (2020). Neuroprotection of Radiosensitive Juvenile Mice by Ultra-High Dose Rate FLASH Irradiation. Cancers.

[130] Fouillade C., Favaudon V., Vozenin M.C., Romeo P.H., Bourhis J., Verrelle P., Devauchelle P., Patriarca A., Heinrich S., Mazal A. (2017). Hopes of high dose-rate radiotherapy. Bull. Cancer.

[131] Chabi S., To T.H.V., Leavitt R., Poglio S., Jorge P.G., Jaccard M., Petersson K., Petit B., Romeo P.H., Pflumio F. (2021). Ultra-high-dose-rate FLASH and Conventional-Dose-Rate Irradiation Differentially Affect Human Acute Lymphoblastic Leukemia and Normal Hematopoiesis. Int. J. Radiat. Oncol. Biol. Phys..

[132] Loo B.W., Schuler E., Lartey F.M., Rafat M., King G.J., Trovati S., Koong A.C., Maxim P.G. (2017). (P003) Delivery of Ultra-Rapid Flash Radiation Therapy and Demonstration of Normal Tissue Sparing After Abdominal Irradiation of Mice. Int. J. Radiat. Oncol. Biol. Phys..

[133] Levy K., Natarajan S., Wang J., Chow S., Eggold J.T., Loo P., Manjappa R., Lartey F.M., Schüler E., Skinner L. (2020). FLASH irradiation enhances the therapeutic index of abdominal radiotherapy for the treatment of ovarian cancer. bioRxiv.

[134] Kacem H., Psoroulas S., Boivin G., Folkerts M., Grilj V., Lomax T., Martinotti A., Meer D., Ollivier J., Petit B. (2022). Comparing radiolytic production of H_2_O_2_ and development of Zebrafish embryos after ultra high dose rate exposure with electron and transmission proton beams. Radiother. Oncol..

[135] Saade G., Bogaerts E., Chiavassa S., Blain G., Delpon G., Evin M., Ghannam Y., Haddad F., Haustermans K., Koumeir C. (2023). Ultrahigh-Dose-Rate Proton Irradiation Elicits Reduced Toxicity in Zebrafish Embryos. Adv. Radiat. Oncol..

[136] Mannerberg A., Konradsson E., Kugele M., Edvardsson A., Kadhim M., Ceberg C., Peterson K., Thomasson H.M., Arendt M.L., Borresen B. (2023). Surface guided electron FLASH radiotherapy for canine cancer patients. Med. Phys..

[137] Michaels H.B., Epp E.R., Ling C.C., Peterson E.C. (1978). Oxygen Sensitization of CHO Cells at Ultrahigh Dose Rates: Prelude to Oxygen Diffusion Studies. Radiat. Res..

[138] Gehart H., Clevers H. (2019). Tales from the crypt: New insights into intestinal stem cells. Nat. Rev. Gastroenterol. Hepatol..

[139] Ruan J.L., Lee C., Wouters S., Tullis I.D.C., Verslegers M., Mysara M., Then C.K., Smart S.C., Hill M.A., Muschel R.J. (2021). Irradiation at Ultra-High (FLASH) Dose Rates Reduces Acute Normal Tissue Toxicity in the Mouse Gastrointestinal System. Int. J. Radiat. Oncol. Biol. Phys..

[140] Makale M.T., McDonald C.R., Hattangadi-Gluth J.A., Kesari S. (2017). Mechanisms of radiotherapy-associated cognitive disability in patients with brain tumours. Nat. Rev. Neurol..

[141] Alaghband Y., Allen B.D., Kramar E.A., Zhang R., Drayson O.G.G., Ru N., Petit B., Almeida A., Doan N.L., Wood M.A. (2023). Uncovering the Protective Neurologic Mechanisms of Hypofractionated FLASH Radiotherapy. Cancer Res. Commun..

[142] Montay-Gruel P., Petit B., Bochud F., Favaudon V., Bourhis J., Vozenin M.C. (2015). PO-0799: Normal brain, neural stem cells and glioblastoma responses to FLASH radiotherapy. Radiother. Oncol..

[143] Allen B.D., Alaghband Y., Kramar E.A., Ru N., Petit B., Grilj V., Petronek M.S., Pulliam C.F., Kim R.Y., Doan N.L. (2023). Elucidating the neurological mechanism of the FLASH effect in juvenile mice exposed to hypofractionated radiotherapy. Neuro Oncol..

[144] Clark R.E., Zola S.M., Squire L.R. (2000). Impaired Recognition Memory in Rats after Damage to the Hippocampus. J. Neurosci..

[145] Oppelt M., Baumann M., Bergmann R., Beyreuther E., Bruchner K., Hartmann J., Karsch L., Krause M., Laschinsky L., Lessmann E. (2015). Comparison study of in vivo dose response to laser-driven versus conventional electron beam. Radiat. Environ. Biophys..

[146] Venkatesulu B.P., Sharma A., Pollard-Larkin J.M., Sadagopan R., Symons J., Neri S., Singh P.K., Tailor R., Lin S.H., Krishnan S. (2019). Ultra high dose rate (35 Gy/sec) radiation does not spare the normal tissue in cardiac and splenic models of lymphopenia and gastrointestinal syndrome. Sci. Rep..

[147] Gaide O., Herrera F., Jeanneret Sozzi W., Gonçalves Jorge P., Kinj R., Bailat C., Duclos F., Bochud F., Germond J.-F., Gondré M. (2022). Comparison of ultra-high versus conventional dose rate radiotherapy in a patient with cutaneous lymphoma. Radiother. Oncol..

[148] Doria D., Kakolee K.F., Kar S., Litt S.K., Fiorini F., Ahmed H., Green S., Jeynes J.C.G., Kavanagh J., Kirby D. (2012). Biological effectiveness on live cells of laser driven protons at dose rates exceeding 109 Gy/s. AIP Adv..

[149] Kim Y.-E., Gwak S.-H., Hong B.-J., Oh J.-M., Choi H.-S., Kim M.S., Oh D., Lartey F.M., Rafat M., Schüler E. (2021). Effects of Ultra-high doserate FLASH Irradiation on the Tumor Microenvironment in Lewis Lung Carcinoma: Role of Myosin Light Chain. Int. J. Radiat. Oncol. Biol. Phys..

[150] Rama N., Saha T., Shukla S., Goda C., Milewski D., Mascia A.E., Vatner R.E., Sengupta D., Katsis A., Abel E. (2019). Improved Tumor Control Through T-cell Infiltration Modulated by Ultra-High Dose Rate Proton FLASH Using a Clinical Pencil Beam Scanning Proton System. Int. J. Radiat. Oncol. Biol. Phys..

[151] Almeida A., Godfroid C., Leavitt R.J., Montay-Gruel P., Petit B., Romero J., Ollivier J., Meziani L., Sprengers K., Paisley R. (2024). Antitumor Effect by Either FLASH or Conventional Dose Rate Irradiation Involves Equivalent Immune Responses. Int. J. Radiat. Oncol. Biol. Phys..

[152] Bogaerts E., Macaeva E., Isebaert S., Haustermans K. (2022). Potential Molecular Mechanisms behind the Ultra-High Dose Rate “FLASH” Effect. Int. J. Mol. Sci..

[153] Mazal A., Prezado Y., Ares C., de Marzi L., Patriarca A., Miralbell R., Favaudon V. (2020). FLASH and minibeams in radiation therapy: The effect of microstructures on time and space and their potential application to protontherapy. Br. J. Radiol..

[154] Durante M., Brauer-Krisch E., Hill M. (2018). Faster and safer? FLASH ultra-high dose rate in radiotherapy. Br. J. Radiol..

[155] Yovino S., Kleinberg L., Grossman S.A., Narayanan M., Ford E. (2013). The Etiology of Treatment-related Lymphopenia in Patients with Malignant Gliomas: Modeling Radiation Dose to Circulating Lymphocytes Explains Clinical Observations and Suggests Methods of Modifying the Impact of Radiation on Immune Cells. Cancer Investig..

[156] Zhu H., Xie D., Wang Y., Huang R., Chen X., Yang Y., Wang B., Peng Y., Wang J., Xiao D. (2023). Comparison of intratumor and local immune response between MV X-ray FLASH and conventional radiotherapies. Clin. Transl. Radiat. Oncol..

[157] Yan O., Wang S., Wang Q., Wang X. (2024). FLASH Radiotherapy: Mechanisms of Biological Effects and the Therapeutic Potential in Cancer. Biomolecules.

[158] Wilson P., Jones B., Yokoi T., Hill M., Vojnovic B. (2012). Revisiting the ultra-high dose rate effect: Implications for charged particle radiotherapy using protons and light ions. Br. J. Radiol..

[159] Adams G.E. (1977). Hypoxic Cell Sensitizers for Radiotherapy. Radiotherapy, Surgery, and Immunotherapy.

[160] Hammond E.M., Asselin M.C., Forster D., O’Connor J.P.B., Senra J.M., Williams K.J. (2014). The Meaning, Measurement and Modification of Hypoxia in the Laboratory and the Clinic. Clin. Oncol..

[161] McKeown S.R. (2014). Defining normoxia, physoxia and hypoxia in tumours—Implications for treatment response. Br. J. Radiol..

[162] Hall E.J. (1972). Radiation Dose-Rate: A Factor of Importance in Radiobiology and Radiotherapy. Br. J. Radiol..

[163] Zhu H., Li J., Deng X., Qiu R., Wu Z., Zhang H. (2021). Modeling of cellular response after FLASH irradiation: A quantitative analysis based on the radiolytic oxygen depletion hypothesis. Phys. Med. Biol..

[164] Cao X., Zhang R., Esipova T.V., Allu S.R., Ashraf R., Rahman M., Gunn J.R., Bruza P., Gladstone D.J., Williams B.B. (2021). Quantification of Oxygen Depletion During FLASH Irradiation In Vitro and In Vivo. Int. J. Radiat. Oncol. Biol. Phys..

[165] Spitz D.R., Buettner G.R., Petronek M.S., St-Aubin J.J., Flynn R.T., Waldron T.J., Limoli C.L. (2019). An integrated physico-chemical approach for explaining the differential impact of FLASH versus conventional dose rate irradiation on cancer and normal tissue responses. Radiother. Oncol..

[166] Dewey D.L. (1969). An Oxygen-Dependent X-Ray Dose-Rate Effect in Serratia marcescens. Radiat. Res..

[167] Ewing D. (2009). Breaking Survival Curves and Oxygen Removal Times in Irradiated Bacterial Spores. Int. J. Radiat. Biol. Relat. Stud. Phys. Chem. Med..

[168] Han J., Mei Z., Lu C., Qian J., Liang Y., Sun X., Pan Z., Kong D., Xu S., Liu Z. (2021). Ultra-High Dose Rate FLASH Irradiation Induced Radio-Resistance of Normal Fibroblast Cells Can Be Enhanced by Hypoxia and Mitochondrial Dysfunction Resulting From Loss of Cytochrome C. Front. Cell Dev. Biol..

[169] Zlobinskaya O., Siebenwirth C., Greubel C., Hable V., Hertenberger R., Humble N., Reinhardt S., Michalski D., Röper B., Multhoff G. (2014). The Effects of Ultra-High Dose Rate Proton Irradiation on Growth Delay in the Treatment of Human Tumor Xenografts in Nude Mice. Radiat. Res..

[170] Wardman P. (2020). Radiotherapy Using High-Intensity Pulsed Radiation Beams (FLASH): A Radiation-Chemical Perspective. Radiat. Res..

[171] Jansen J., Beyreuther E., Garcia-Calderon D., Karsch L., Knoll J., Pawelke J., Schurer M., Seco J. (2022). Changes in Radical Levels as a Cause for the FLASH effect: Impact of beam structure parameters at ultra-high dose rates on oxygen depletion in water. Radiother. Oncol..

[172] Hu A., Qiu R., Li W.B., Zhou W., Wu Z., Zhang H., Li J. (2022). Radical recombination and antioxidants: A hypothesis on the FLASH effect mechanism. Int. J. Radiat. Biol..

[173] Labarbe R., Hotoiu L., Barbier J., Favaudon V. (2020). A physicochemical model of reaction kinetics supports peroxyl radical recombination as the main determinant of the FLASH effect. Radiother. Oncol..

[174] Hanahan D., Weinberg R.A. (2011). Hallmarks of Cancer: The Next Generation. Cell.

[175] Aykin-Burns N., Ahmad I.M., Zhu Y., Oberley L.W., Spitz D.R. (2009). Increased levels of superoxide and H2O2 mediate the differential susceptibility of cancer cells versus normal cells to glucose deprivation. Biochem. J..

[176] Blain G., Vandenborre J., Villoing D., Fiegel V., Fois G.R., Haddad F., Koumeir C., Maigne L., Métivier V., Poirier F. (2022). Proton Irradiations at Ultra-High Dose Rate vs. Conventional Dose Rate: Strong Impact on Hydrogen Peroxide Yield. Radiat. Res..

[177] Asaithamby A., Hu B., Delgado O., Ding L.H., Story M.D., Minna J.D., Shay J.W., Chen D.J. (2011). Irreparable complex DNA double-strand breaks induce chromosome breakage in organotypic three-dimensional human lung epithelial cell culture. Nucleic Acids Res..

[178] Osipov A., Chigasova A., Yashkina E., Ignatov M., Fedotov Y., Molodtsova D., Vorobyeva N., Osipov A.N. (2023). Residual Foci of DNA Damage Response Proteins in Relation to Cellular Senescence and Autophagy in X-Ray Irradiated Fibroblasts. Cells.

[179] Bushmanov A., Vorobyeva N., Molodtsova D., Osipov A.N. (2022). Utilization of DNA double-strand breaks for biodosimetry of ionizing radiation exposure. Environ. Adv..

[180] Osipov A., Chigasova A., Yashkina E., Ignatov M., Vorobyeva N., Zyuzikov N., Osipov A.N. (2024). Early and Late Effects of Low-Dose X-ray Exposure in Human Fibroblasts: DNA Repair Foci, Proliferation, Autophagy, and Senescence. Int. J. Mol. Sci..

[181] Schmid T.E., Dollinger G., Hable V., Greubel C., Zlobinskaya O., Michalski D., Molls M., Röper B. (2010). Relative biological effectiveness of pulsed and continuous 20MeV protons for micronucleus induction in 3D human reconstructed skin tissue. Radiother. Oncol..

[182] Zhou G. (2020). Mechanisms underlying FLASH radiotherapy, a novel way to enlarge the differential responses to ionizing radiation between normal and tumor tissues. Radiat. Med. Prot..

[183] Cooper C.R., Jones D., Jones G.D.D., Petersson K. (2022). FLASH irradiation induces lower levels of DNA damage ex vivo, an effect modulated by oxygen tension, dose, and dose rate. Br. J. Radiol..

[184] Hageman E., Che P.P., Dahele M., Slotman B.J., Sminia P. (2022). Radiobiological Aspects of FLASH Radiotherapy. Biomolecules.

[185] Babayan N., Vorobyeva N., Grigoryan B., Grekhova A., Pustovalova M., Rodneva S., Fedotov Y., Tsakanova G., Aroutiounian R., Osipov A. (2020). Low Repair Capacity of DNA Double-Strand Breaks Induced by Laser-Driven Ultrashort Electron Beams in Cancer Cells. Int. J. Mol. Sci..

[186] Asaithamby A., Hu B., Chen D.J. (2011). Unrepaired clustered DNA lesions induce chromosome breakage in human cells. Proc. Natl. Acad. Sci. USA.

[187] Carter R.J., Nickson C.M., Thompson J.M., Kacperek A., Hill M.A., Parsons J.L. (2018). Complex DNA Damage Induced by High Linear Energy Transfer Alpha-Particles and Protons Triggers a Specific Cellular DNA Damage Response. Int. J. Radiat. Oncol. Biol. Phys..

[188] Lanz M.C., Dibitetto D., Smolka M.B. (2019). DNA damage kinase signaling: Checkpoint and repair at 30 years. EMBO J..

[189] Weinert T.A., Hartwell L.H. (1988). The RAD9 Gene Controls the Cell Cycle Response to DNA Damage in Saccharomyces cerevisiae. Science.

[190] Shibata A., Jeggo P.A. (2014). DNA Double-strand Break Repair in a Cellular Context. Clin. Oncol..

[191] Belov O., Chigasova A., Pustovalova M., Osipov A., Eremin P., Vorobyeva N., Osipov A.N. (2023). Dose-Dependent Shift in Relative Contribution of Homologous Recombination to DNA Repair after Low-LET Ionizing Radiation Exposure: Empirical Evidence and Numerical Simulation. Curr. Issues Mol. Biol..

[192] Chang L., Hu W., Ye C., Yao B., Song L., Wu X., Ding N., Wang J., Zhou G. (2014). miR-3928 activates ATR pathway by targeting Dicer. RNA Biol..

[193] Wang J., He J., Su F., Ding N., Hu W., Yao B., Wang W., Zhou G. (2013). Repression of ATR pathway by miR-185 enhances radiation-induced apoptosis and proliferation inhibition. Cell Death Dis..

[194] Su F., Smilenov L.B., Ludwig T., Zhou L., Zhu J., Zhou G., Hall E.J. (2010). Hemizygosity for Atm and Brca1 influence the balance between cell transformation and apoptosis. Radiat. Oncol..

[195] Farres J., Martin-Caballero J., Martinez C., Lozano J.J., Llacuna L., Ampurdanes C., Ruiz-Herguido C., Dantzer F., Schreiber V., Villunger A. (2013). Parp-2 is required to maintain hematopoiesis following sublethal gamma-irradiation in mice. Blood.

[196] Malanga M., Althaus F.R. (2006). DNA Damage Signaling through Poly(ADP-Ribose). Poly(ADP-Ribosyl)ation.

[197] Lonn P., van der Heide L.P., Dahl M., Hellman U., Heldin C.H., Moustakas A. (2010). PARP-1 attenuates Smad-mediated transcription. Mol. Cell.

[198] Scharpfenecker M., Kruse J.J., Sprong D., Russell N.S., Ten Dijke P., Stewart F.A. (2009). Ionizing radiation shifts the PAI-1/ID-1 balance and activates notch signaling in endothelial cells. Int. J. Radiat. Oncol. Biol. Phys..

[199] Gluck S., Guey B., Gulen M.F., Wolter K., Kang T.W., Schmacke N.A., Bridgeman A., Rehwinkel J., Zender L., Ablasser A. (2017). Innate immune sensing of cytosolic chromatin fragments through cGAS promotes senescence. Nat. Cell Biol..

[200] Ruiz de Galarreta M., Lujambio A. (2017). DNA sensing in senescence. Nat. Cell Biol..

[201] Vanpouille-Box C., Alard A., Aryankalayil M.J., Sarfraz Y., Diamond J.M., Schneider R.J., Inghirami G., Coleman C.N., Formenti S.C., Demaria S. (2017). DNA exonuclease Trex1 regulates radiotherapy-induced tumour immunogenicity. Nat. Commun..

[202] Yang H., Wang H., Ren J., Chen Q., Chen Z.J. (2017). cGAS is essential for cellular senescence. Proc. Natl. Acad. Sci. USA.

[203] Wu S., Zhang Q., Zhang F., Meng F., Liu S., Zhou R., Wu Q., Li X., Shen L., Huang J. (2019). HER2 recruits AKT1 to disrupt STING signalling and suppress antiviral defence and antitumour immunity. Nat. Cell Biol..

[204] Catalano F., Borea R., Puglisi S., Boutros A., Gandini A., Cremante M., Martelli V., Sciallero S., Puccini A. (2022). Targeting the DNA Damage Response Pathway as a Novel Therapeutic Strategy in Colorectal Cancer. Cancers.

[205] Lozano R., Castro E., Aragón I.M., Cendón Y., Cattrini C., López-Casas P.P., Olmos D. (2020). Genetic aberrations in DNA repair pathways: A cornerstone of precision oncology in prostate cancer. Br. J. Cancer.

[206] Kumar V., Bauer C., Stewart J.H.t. (2023). Cancer cell-specific cGAS/STING Signaling pathway in the era of advancing cancer cell biology. Eur. J. Cell Biol..

[207] Johnston C.J., Williams J.P., Okunieff P., Finkelstein J.N. (2002). Radiation-Induced Pulmonary Fibrosis: Examination of Chemokine and Chemokine Receptor Families. Radiat. Res..

[208] Missiroli S., Genovese I., Perrone M., Vezzani B., Vitto V.A.M., Giorgi C. (2020). The Role of Mitochondria in Inflammation: From Cancer to Neurodegenerative Disorders. J. Clin. Med..

[209] West A.P., Shadel G.S. (2017). Mitochondrial DNA in innate immune responses and inflammatory pathology. Nat. Rev. Immunol..

[210] Marchi S., Giorgi C., Suski J.M., Agnoletto C., Bononi A., Bonora M., De Marchi E., Missiroli S., Patergnani S., Poletti F. (2012). Mitochondria-Ros Crosstalk in the Control of Cell Death and Aging. J. Signal Transduct..

[211] Guo Z., Buonanno M., Harken A., Zhou G., Hei T.K. (2022). Mitochondrial Damage Response and Fate of Normal Cells Exposed to FLASH Irradiation with Protons. Radiat. Res..

[212] Rongvaux A., Jackson R., Harman C.C.D., Li T., West A.P., de Zoete M.R., Wu Y., Yordy B., Lakhani S.A., Kuan C.-Y. (2014). Apoptotic Caspases Prevent the Induction of Type I Interferons by Mitochondrial DNA. Cell.

[213] Lv J., Sun J., Luo Y., Liu J., Wu D., Fang Y., Lan H., Diao L., Ma Y., Li Y. (2024). FLASH Irradiation Regulates IFN-β induction by mtDNA via Cytochrome c Leakage. bioRxiv.

[214] Warburg O., Wind F., Negelein E. (1927). The Metabolism of Tumors in the Body. J. Gen. Physiol..

[215] Zhang Y., Ding Z., Perentesis J.P., Khuntia D., Pfister S.X., Sharma R.A. (2021). Can Rational Combination of Ultra-high Dose Rate FLASH Radiotherapy with Immunotherapy Provide a Novel Approach to Cancer Treatment?. Clin. Oncol..

[216] Herrera F.G., Bourhis J., Coukos G. (2016). Radiotherapy combination opportunities leveraging immunity for the next oncology practice. CA Cancer J. Clin..

[217] Bristow R.G., Alexander B., Baumann M., Bratman S.V., Brown J.M., Camphausen K., Choyke P., Citrin D., Contessa J.N., Dicker A. (2018). Combining precision radiotherapy with molecular targeting and immunomodulatory agents: A guideline by the American Society for Radiation Oncology. Lancet Oncol..

[218] Sharma S., Narayanasamy G., Przybyla B., Webber J., Boerma M., Clarkson R., Moros E.G., Corry P.M., Griffin R.J. (2016). Advanced Small Animal Conformal Radiation Therapy Device. Technol. Cancer Res. Treat..

[219] Verhaegen F., Dubois L., Gianolini S., Hill M.A., Karger C.P., Lauber K., Prise K.M., Sarrut D., Thorwarth D., Vanhove C. (2018). ESTRO ACROP: Technology for precision small animal radiotherapy research: Optimal use and challenges. Radiother. Oncol..

[220] Keall P.J., Mageras G.S., Balter J.M., Emery R.S., Forster K.M., Jiang S.B., Kapatoes J.M., Low D.A., Murphy M.J., Murray B.R. (2006). The management of respiratory motion in radiation oncology report of AAPM Task Group 76a). Med. Phys..

[221] Almond P.R., Biggs P.J., Coursey B.M., Hanson W.F., Huq M.S., Nath R., Rogers D.W. (1999). AAPM’s TG-51 protocol for clinical reference dosimetry of high-energy photon and electron beams. Med. Phys..

[222] Karsch L., Beyreuther E., Burris-Mog T., Kraft S., Richter C., Zeil K., Pawelke J. (2012). Dose rate dependence for different dosimeters and detectors: TLD, OSL, EBT films, and diamond detectors. Med. Phys..

[223] Wei S., Shi C., Chen C.-C., Huang S., Press R.H., Choi J.I., Simone Ii C.B., Lin H., Kang M. (2022). Recent progress in pencil beam scanning FLASH proton therapy: A narrative review. Ther. Radiol. Oncol..

[224] Miles D., Sforza D., Wong J., Rezaee M. (2024). Dosimetric characterization of a rotating anode X-ray tube for FLASH radiotherapy research. Med. Phys..

[225] Hartsell W.F., Simone C.B., Godes D., Maggiore J., Mehta M.P., Frank S.J., Metz J.M., Choi J.I. (2024). Temporal Evolution and Diagnostic Diversification of Patients Receiving Proton Therapy in the United States: A Ten-Year Trend Analysis (2012 to 2021) From the National Association for Proton Therapy. Int. J. Radiat. Oncol. Biol. Phys..

[226] Abel E., Girdhani S., Jackson I., Eley J., Katsis A., Marshall A., Rodriguez A., Senapati S., Bentzen S.M., Vujaskovic Z. (2019). Characterization of Radiation-Induced Lung Fibrosis and Mode of Cell Death Using Single and Multi-Pulsed Proton Flash Irradiation. Int. J. Radiat. Oncol. Biol. Phys..

[227] Pawelke J., Brand M., Hans S., Hideghety K., Karsch L., Lessmann E., Lock S., Schurer M., Szabo E.R., Beyreuther E. (2021). Electron dose rate and oxygen depletion protect zebrafish embryos from radiation damage. Radiother. Oncol..

[228] Eggold J.T., Chow S., Melemenidis S., Wang J., Natarajan S., Loo P.E., Manjappa R., Viswanathan V., Kidd E.A., Engleman E. (2022). Abdominopelvic FLASH Irradiation Improves PD-1 Immune Checkpoint Inhibition in Preclinical Models of Ovarian Cancer. Mol. Cancer Ther..

[229] Sørensen B.S., Sitarz M.K., Ankjærgaard C., Johansen J.G., Andersen C.E., Kanouta E., Grau C., Poulsen P. (2022). Pencil beam scanning proton FLASH maintains tumor control while normal tissue damage is reduced in a mouse model. Radiother. Oncol..

[230] Tinganelli W., Weber U., Puspitasari A., Simoniello P., Abdollahi A., Oppermann J., Schuy C., Horst F., Helm A., Fournier C. (2022). FLASH with carbon ions: Tumor control, normal tissue sparing, and distal metastasis in a mouse osteosarcoma model. Radiother. Oncol..

[231] Tinganelli W., Sokol O., Quartieri M., Puspitasari A., Dokic I., Abdollahi A., Durante M., Haberer T., Debus J., Boscolo D. (2022). Ultra-High Dose Rate (FLASH) Carbon Ion Irradiation: Dosimetry and First Cell Experiments. Int. J. Radiat. Oncol. Biol. Phys..

[232] Auer S., Hable V., Greubel C., Drexler G.A., Schmid T.E., Belka C., Dollinger G., Friedl A.A. (2011). Survival of tumor cells after proton irradiation with ultra-high dose rates. Radiat. Oncol..

[233] Adrian G., Konradsson E., Beyer S., Wittrup A., Butterworth K.T., McMahon S.J., Ghita M., Petersson K., Ceberg C. (2021). Cancer Cells Can Exhibit a Sparing FLASH Effect at Low Doses Under Normoxic In Vitro-Conditions. Front. Oncol..

[234] Boscolo D., Scifoni E., Durante M., Kramer M., Fuss M.C. (2021). May oxygen depletion explain the FLASH effect? A chemical track structure analysis. Radiother. Oncol..

[235] Montay-Gruel P., Markarian M., Allen B.D., Baddour J.D., Giedzinski E., Jorge P.G., Petit B., Bailat C., Vozenin M.C., Limoli C. (2020). Ultra-High-Dose-Rate FLASH Irradiation Limits Reactive Gliosis in the Brain. Radiat. Res..

